# Association of perioperative P2Y_12_ inhibitor administration with outcomes for tandem occlusion: RESCUE AT-LVO sub-study

**DOI:** 10.3389/fneur.2024.1475882

**Published:** 2024-11-21

**Authors:** Takeshi Yoshimoto, Hiroshi Yamagami, Nobuyuki Sakai, Kazutaka Uchida, Manabu Shirakawa, Mikiya Beppu, Kazunori Toyoda, Yuji Matsumaru, Yasushi Matsumoto, Kenichi Todo, Mikito Hayakawa, Seigo Shindo, Masafumi Morimoto, Masataka Takeuchi, Hirotoshi Imamura, Hiroyuki Ikeda, Kanta Tanaka, Hideyuki Ishihara, Hiroto Kakita, Takanori Sano, Hayato Araki, Tatsufumi Nomura, Fumihiro Sakakibara, Shinichi Yoshimura, for RESCUE AT-LVO Investigators

**Affiliations:** ^1^Department of Stroke and Cerebrovascular Diseases, University of Tsukuba Hospital, Tsukuba, Japan; ^2^Department of Neurology, National Cerebral and Cardiovascular Center, Suita, Japan; ^3^Division of Stroke Prevention and Treatment, Institute of Medicine, University of Tsukuba, Tsukuba, Japan; ^4^Department of Stroke Neurology, NHO Osaka National Hospital, Osaka, Japan; ^5^Department of Neurosurgery, Seijinkai Shimizu Hospital, Kyoto, Japan; ^6^Department of Neurosurgery, Hyogo Medical University, Nishinomiya, Japan; ^7^Department of Cerebrovascular Medicine, National Cerebral and Cardiovascular Center, Suita, Japan; ^8^Department of Neurosurgery, Institute of Medicine, University of Tsukuba, Tsukuba, Japan; ^9^Division of Development and Discovery of Interventional Therapy, Tohoku University Hospital, Sendai, Japan; ^10^Stroke Center, Osaka University Graduate School of Medicine, Suita, Japan; ^11^Department of Neurology, Institute of Medicine, University of Tsukuba, Tsukuba, Japan; ^12^Department of Neurology, Japanese Red Cross Kumamoto Hospital, Kumamoto, Japan; ^13^Department of Neurosurgery, Yokohama Shintoshi Neurosurgical Hospital, Yokohama, Japan; ^14^Department of Neurosurgery, Seisho Hospital, Odawara, Japan; ^15^Department of Neurosurgery, National Cerebral and Cardiovascular Center, Suita, Japan; ^16^Department of Neurosurgery, Kurashiki Central Hospital, Kurashiki, Japan; ^17^Stroke Center, Kindai University Hospital, Sayama, Japan; ^18^Department of Neurosurgery, Yamaguchi University School of Medicine, Ube, Japan; ^19^Department of Neurosurgery, Japanese Red Cross Ise Hospital, Ise, Japan; ^20^Department of Neurosurgery, Mie Prefectural General Medical Center, Yokkaichi, Japan; ^21^Department of Neurosurgery, Araki Neurosurgical Hospital, Hiroshima, Japan; ^22^Department of Neurosurgery, Ohkawara Neurosurgical Hospital, Muroran, Japan

**Keywords:** stroke, tandem occlusion, P2Y12 inhibitor, endovascular therapy, carotid artery stenting

## Abstract

**Background:**

We aimed to clarify the association between intraoperative P2Y_12_ inhibitor administration during EVT and clinical outcomes in patients with anterior circulation TO stroke.

**Methods:**

Among consecutive patients with acute ischemic stroke (AIS) enrolled in the Recovery by Endovascular Salvage for Cerebral Ultra-acute Embolic and Atherothrombotic Stroke with Large Vessel Occlusion Registry from 2016 to 2019, those with anterior circulation TOs who underwent EVT were analyzed. These patients were categorized into the following groups: those who received P2Y_12_ inhibitors during the perioperative period and those who did not receive P2Y_12_ inhibitors. The outcomes included good functional outcomes, as indicated by a modified Rankin Scale score of 0–2 at 90 days, and the incidence of symptomatic intracranial hemorrhage (SICH) was compared between the two groups. Multivariate logistic regression models were used to assess the association of outcomes with perioperative P2Y_12_ inhibitor administration. Odds ratios (ORs) with 95% confidence intervals (CIs) were calculated using the group that did not receive P2Y_12_ inhibitors as the reference. The perioperative period included the period in which antithrombotic therapy was administered immediately before EVT and during the operative period.

**Results:**

We enrolled 242 patients with AIS with anterior circulation TOs (42 females [17.4%]; median age, 76 [interquartile range, 69–81] years). Patients who received P2Y_12_ inhibitors during the perioperative period (*n* = 131) showed a higher frequency of carotid artery stenting than those who did not receive perioperative P2Y_12_ inhibitors (*n* = 111; 86.3% vs. 42.3%, *p* < 0.01). Furthermore, patients who received perioperative P2Y_12_ inhibitors during the perioperative period had a higher incidence of good functional outcomes than those who did not receive perioperative P2Y_12_ inhibitors (42.0% vs. 32.4%; adjusted OR: 6.65, 95% CI: 1.88–23.53), with no significant differences between the groups in the incidence of SICH (5.3% vs. 8.1%; OR: 0.44; 95% CI: 0.09–2.09).

**Conclusion:**

Perioperative administration of P2Y_12_ inhibitors may be associated with a higher frequency of good functional outcomes in patients undergoing EVT for AIS with anterior circulation TOs. However, since several confounding factors are involved in this sub-analysis of EVT for anterior circulation TOs, further studies are warranted.

## 1 Introduction

Several unanswered questions remain regarding the optimal perioperative antithrombotic management of tandem occlusions (TOs), which are characterized by the coexistence of a cervical internal carotid artery (c-ICA) occlusion or high-grade stenosis and an ipsilateral large intracranial vessel occlusion (internal carotid artery [ICA] or middle cerebral artery [MCA] M1/M2) (1, 2), with antithrombotic treatment for acute ischemic stroke (IS) due to TOs of the anterior circulation being particularly controversial due to the lack of randomized controlled trials (RCTs) evaluating its effectiveness and safety (2). Currently, no guidelines or recommendations exist based on high-quality evidence for optimal antithrombotic treatment of patients with acute IS (AIS) due to TOs undergoing endovascular therapy (EVT), including carotid artery stenting (CAS) because three major EVT RCTs (3–5) excluded these patients; the remaining major EVT RCTs enrolled relatively few patients with TOs, representing 13–32% (6). Therefore, the American Heart Association/American Stroke Association guideline (7) and the European Stroke Organization guidelines (8) do not specify the optimal antithrombotic therapy for TO. Currently, general antithrombotic treatment recommendations suggest several options for antithrombotic therapy in TO, including no antiplatelet agent, single antiplatelet therapy (SAPT), dual antiplatelet therapy (DAPT), or glycoprotein IIb/IIIa inhibitor; however, no established consensus exist on the best approach. DAPT is used as the standard of care during the perioperative period for CAS in real-world practice because it results in fewer ischemic and hemorrhagic complications than anticoagulant therapy (9).

Antiplatelet therapy (APT) administered pre-treatment reduces procedural embolic events and re-occlusion of c-ICA lesions (10, 11), and APT during EVT for anterior circulation TO is safe and associated with lower 90-day mortality (12). However, other studies have reported that antithrombotic therapy during acute stenting increases the risk of symptomatic intracranial hemorrhage (SICH) (13). The risk–benefit balance of introducing APT during EVT for TO is still under debate. Furthermore, a previous study showed that no difference was found in the rate of good outcomes or the incidence of bleeding complications between DAPT with aspirin and P2Y_12_ inhibitors (clopidogrel, ticagrelor, and prasugrel) and SAPT with aspirin alone in the perioperative period of EVT for acute anterior circulation TO (14). A previous meta-analysis of RCTs on atherosclerotic cardiovascular disease showed that P2Y_12_ inhibitor monotherapy was associated with a significant reduction in atherothrombotic events without increasing the risk of major bleeding compared with aspirin alone (15). However, data on the clinical outcomes and safety of P2Y_12_ inhibitor administration during EVT for TOs are limited (16). Therefore, this study aimed to investigate the clinical outcomes and safety of P2Y_12_ inhibitor administration during the perioperative period of EVT for anterior circulation TOs in a large Japanese multicenter cohort.

## 2 Materials and methods

### 2.1 Ethics statement

Clinical data were collected at each hospital through chart review or contact with patients or relatives. This study was conducted in accordance with the Declaration of Helsinki and conformed to Strengthening the Reporting of Observational Studies in Epidemiology (STROBE) guidelines (17). The complete STROBE checklist is included in Supplementary material. Furthermore, the requirement for written informed consent was waived because the study was retrospective and used anonymized data.

### 2.2 Study participants

All patients with AIS due to large vessel occlusion (LVO) caused by intracranial atherosclerosis or extracranial carotid atherosclerosis admitted within 7 days of the last known well (LKW) were retrospectively registered in the Recovery by Endovascular Salvage for Cerebral Ultra-acute Embolic and Atherothrombotic Stroke with Large Vessel Occlusion (RESCUE AT-LVO) (18, 19), a historical multicenter registry that included data from 51 hospitals in Japan from January 2017 to December 2019.

For the present sub-study, we reviewed the findings of consecutive patients enrolled in this registry who met the following criteria: (1) AIS due to anterior circulation LVO of the extracranial or intracranial ICA and the M1 or M2 segment of the MCA; (2) underwent EVT; (3) showed TOs (occlusion or stenosis at the c-ICA with ipsilateral intracranial artery occlusion); and (4) available for modified Rankin Scale (mRS) score at 90 days. Patients were excluded if they met any of the following criteria: (1) stenosis caused by a non-atherosclerotic etiology, such as moyamoya disease, arterial dissection, or vasculitis; (2) multiple acute infarctions in multiple vascular territories, excluding artery-to-artery embolism due to c-ICA occlusion or stenosis; (3) underwent EVT alone for cervical lesions; (4) had an unknown onset time; or (5) had posterior circulation LVO.

### 2.3 Clinical data collection

The following clinical data were collected: age, sex, pre-stroke mRS score, baseline systolic blood pressure, baseline National Institutes of Health Stroke Scale (NIHSS) score, medical history (atrial fibrillation, hypertension, diabetes mellitus, dyslipidemia, IS/transit ischemic attack prior to index stroke, and ischemic heart disease), antithrombotic drugs prior to index stroke (any antiplatelet drugs, single antiplatelet drug, dual antiplatelet drugs, any anticoagulant drugs, and statins), statin use prior to index stroke, and imaging (the Alberta Stroke Program Early Computed Tomographic Score [ASPECTS] on diffusion-weighted magnetic resonance imaging [MRI] or non-contrast computed tomography [CT]). Procedural variables included details of thrombectomy (stent retriever/combined contact aspiration and stent retrievers, contact aspiration, angioplasty, and CAS), antegrade/retrograde thrombectomy, and additional antithrombotic medication during the perioperative period (aspirin, clopidogrel, cilostazol, ticagrelor, prasugrel, intravenous ozagrel sodium, warfarin, and direct oral anticoagulants). Imaging findings included the presence of c-ICA lesions (c-ICA occlusion and stenosis) and the degree of stenosis of the cervical lesion at baseline according to the North American Symptomatic Carotid Endarterectomy Trial (20). Intravenous thrombolysis was performed using alteplase (0.6 mg/kg: the dose approved in Japan) (21). Time delays included time from LKW to hospital arrival, time from LKW to groin puncture, and time from groin puncture to modified Thrombolysis In Cerebral Infarction scale (mTICI) ≥2a reperfusion. Procedural variables included details regarding thrombectomy, antegrade/retrograde thrombectomy, and additional antithrombotic medications during the perioperative period. The perioperative period included the period in which antithrombotic therapy was administered immediately before thrombectomy and during the operative period.

### 2.4 Endovascular therapy

All EVT procedures were performed by physicians certified by the Japanese Society for Neuroendovascular Therapy (22), as recommended by the American Heart Association/American Stroke Association guidelines (7) and the guidelines from the Japan Stroke Society, the Japan Neurological Society, and the Japanese Society for Neuroendovascular Therapy (22). EVT procedures included stent retriever/combined contact aspiration, stent retriever application (23), contact aspiration, angioplasty, and CAS. Procedural device selection was at the discretion of the treating physician, although limited to those approved in Japan. Additionally, the decision to perform antegrade or retrograde thrombectomy or angioplasty/CAS for TO was made at the physician’s discretion. The reperfusion status after EVT was assessed using the mTICI (24).

### 2.5 Antiplatelet strategies during EVT

The type and dosage of APT regimens in the perioperative period (preoperative and intraoperative) were determined by the treating physician according to the institution’s protocol and included aspirin, P2Y_12_ inhibitors (clopidogrel or prasugrel), and/or cilostazol. Since glycoprotein (GP) IIB/III A inhibitors (tirofiban, epifibatide, or abciximab) and other P2Y_12_ inhibitors (ticagrelor and cangrelor) are not approved in Japan for ischemic stroke, they were excluded from this sub-study. In this study, DAPT was defined as APT with any two of aspirin, clopidogrel, cilostazol, or prasugrel, and triple APT (TAPT) was defined as APT with any three of aspirin, clopidogrel, cilostazol, or prasugrel. Based on the results of the PRASTRO integrated study (25), prasugrel was approved in Japan in December 2021 for the treatment of non-cardioembolic ischemic stroke within 7 days of onset.

### 2.6 Outcomes

The primary outcome was an mRS score of 0–2 at 90 days, indicating a good functional outcome. Secondary outcomes were defined as death within 90 days, any hemorrhagic event, any intracranial hemorrhage (ICH), SICH, any ischemic event, recurrent IS, post-procedure re-occlusion, and mRS shifts (an increase of 1 point in the mRS score). ICH was assessed using non-contrast CT or gradient-echo MRI 24 ± 8 h after the procedure. Any ICH was defined as any new ICH on imaging, irrespective of the symptoms. SICH was defined as any ICH with a ≥ 4-point increase in the NIHSS score from baseline according to the Heidelberg classification (26). Procedural outcomes were final mTICI ≥2b reperfusion, final mTICI ≥2c reperfusion, and re-occlusion during the procedure.

### 2.7 Statistical analysis

Data were summarized as median (interquartile range [IQR]) for continuous variables and frequencies and percentages for categorical variables. The number of missing observed variables is also presented. Patients were categorized into those who received a P2Y_12_ inhibitor (clopidogrel or prasugrel) in the perioperative period (P2Y_12_ inhibitor [+] group) and those who did not (P2Y_12_ inhibitor (−) group). Statistically significant differences between groups were assessed using the Mann–Whitney *U* test, Student’s t-test, Wilcoxon rank-sum test, χ2 test, or Fisher’s exact test, as appropriate. Multivariate logistic regression models were used to evaluate the association between primary and secondary outcomes and P2Y_12_ inhibitor administration in the perioperative period. Odds ratios (ORs) with 95% confidence intervals (CIs) were calculated using the P2Y_12_ inhibitor (−) group as a reference. The following prespecified variables were included: sex (27), age (28–30), pre-stroke mRS (30), baseline NIHSS score (27), atrial fibrillation (31), hypertension (32), diabetes mellitus (33), dyslipidemia (34), ASPECTS (35), intravenous thrombolysis (28), statin use (36), aspirin administration during the perioperative period (37), and angioplasty and CAS (38). An ordinal logistic regression model was used to analyze shifts in mRS scores at 90 days. Regarding sensitivity analysis, we used inverse probability of treatment weighting (IPTW) to adjust for differences in baseline characteristics. The propensity scores of IPTW analyses were calculated using a mixed-effect logistic regression model. Patients with anterior circulation TO were classified as having occlusion or stenosis of the c-ICA with ipsilateral intracranial artery occlusion, and the same analysis was performed for each group with or without perioperative P2Y_12_ inhibitors. Moreover, the same analysis was performed for patients who underwent CAS or those who received perioperative aspirin. Patients were categorized into four groups (no APT, SAPT, DAPT, and TAPT) according to the number of antiplatelet drugs administered in the perioperative period, and patient backgrounds and outcomes were compared. All analyses were performed using the Stata/IC statistical package, version 17.1 (Stata Corp LLC, College Station, TX, United States).

## 3 Results

### 3.1 Patient characteristics

**Figure 1:**
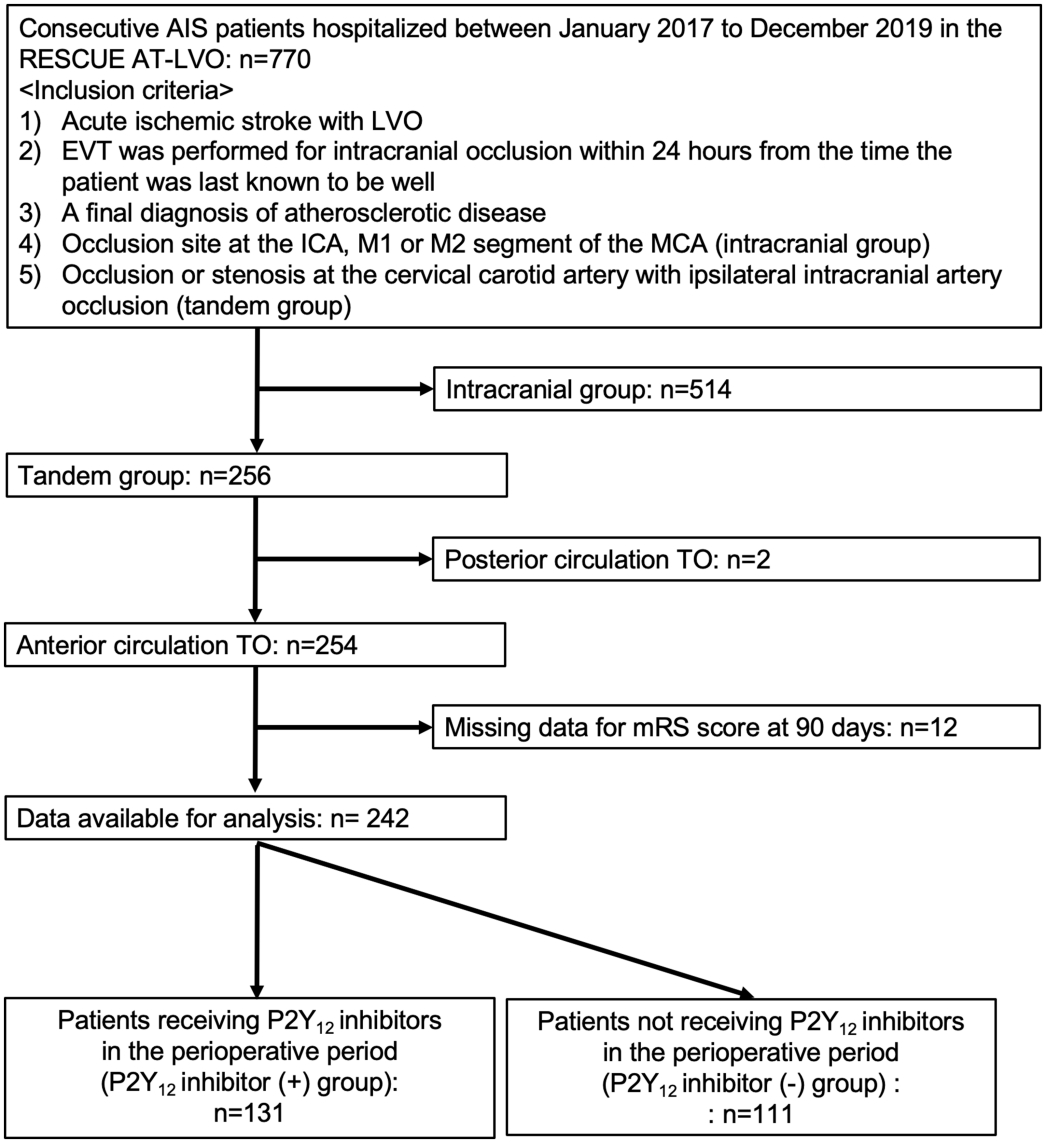
Study flow chart. AIS, acute ischemic stroke; RESCUE AT-LVO, Recovery by Endovascular Salvage for Cerebral Ultra-acute Embolic and Atherothrombotic Stroke with Large Vessel Occlusion; TO, tandem occlusions; ICA, internal carotid artery; MCA; LVO, large-vessel occlusion; mRS, modified Rankin Scale.

Among the 770 patients undergoing EVT for AIS due to extracranial carotid atherosclerosis with anterior circulation TOs enrolled in the RESCUE AT-LVO registry, after excluding 514 with intracranial atherosclerotic stenosis-related LVO stroke, 2 with acute posterior-circulation TOs, and 12 with missing mRS scores at 90 days, the remaining 242 patients with acute anterior circulation TOs (42 females [17.4%]; median age, 76 years [IQR, 69–81 years]; median NIHSS score, 15 [IQR, 10–21]) who underwent EVT were analyzed in this study (Figure 1).

**Figure 2:**
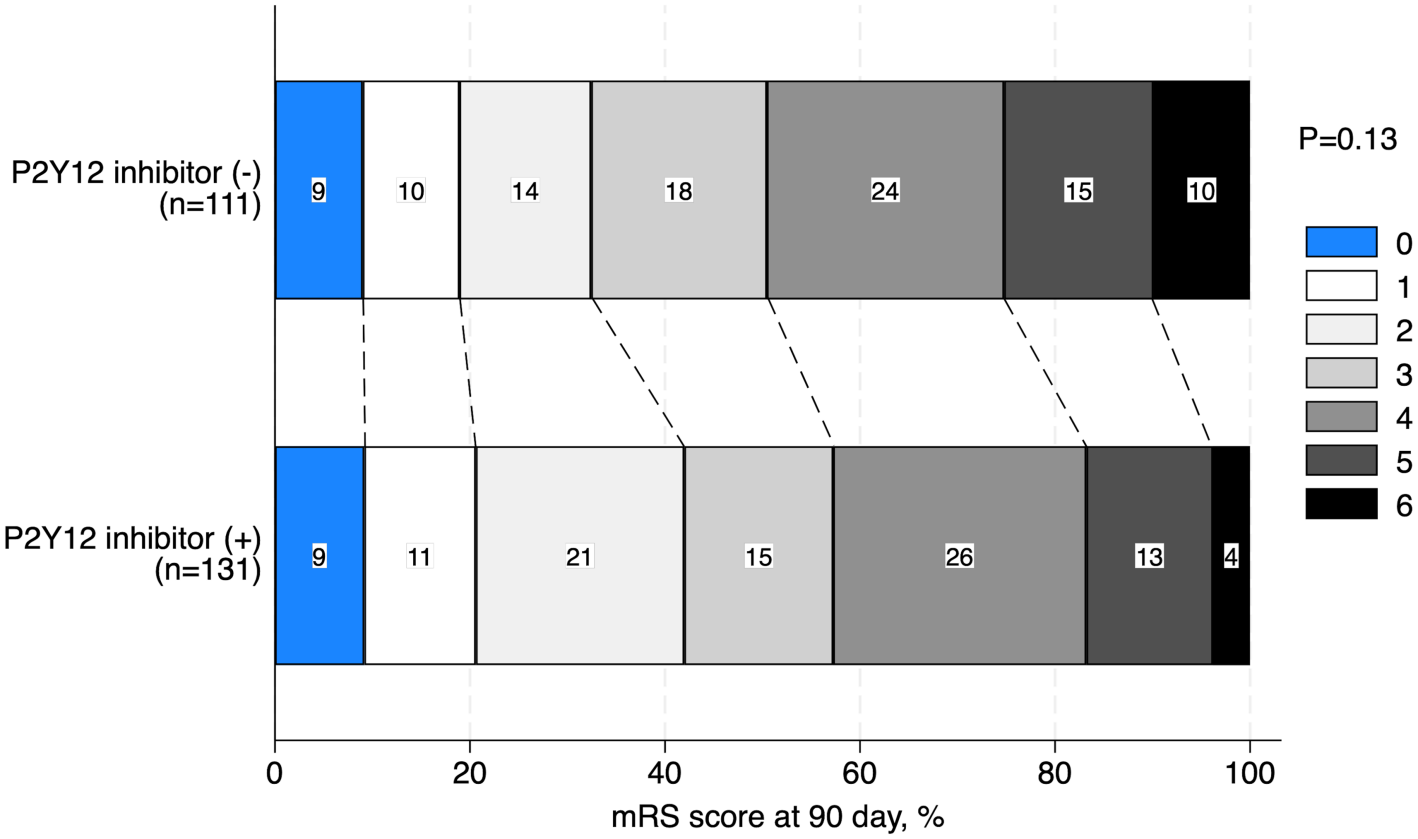
Distribution of the mRS score at 90 days. mRS, modified Rankin Scale.

The baseline characteristics of the patients with TOs treated with and without P2Y_12_ inhibitors during the perioperative period are shown in Table 1. Patients who received P2Y_12_ inhibitors in the perioperative period showed a lower frequency of atrial fibrillation (1.5% vs. 8.1%, *p* = 0.03), any anticoagulant drugs prior to the index stroke (1.5% vs. 9.0%, *p* = 0.01), and perioperative administration with cilostazol (3.8% vs. 10.8%, *p* < 0.01), and a higher frequency of CAS (86.3% vs. 42.3%, *p* < 0.01) and treatment with aspirin during the perioperative period than those who did not receive P2Y_12_ inhibitors (93.1% vs. 20.7%, *p* < 0.01). The two groups showed no significant differences in procedural complications during EVT (Table 2). Patients with TO treated with P2Y_12_ inhibitors in the perioperative period showed a higher frequency of good functional outcomes than those who were not treated with P2Y_12_ inhibitors (mRS scores of 0–2 at 90 days; 42.0% vs. 32.4%; adjusted OR: 4.08, 95% CI: 1.31–12.63). The two groups showed no significant difference in the incidence of death within 90 days (3.8% vs. 9.9%; adjusted OR: 0.82, 95% CI: 0.14–4.90), any ICH (9.2% vs. 15.3%; adjusted OR: 0.33, 95% CI: 0.09–1.06), SICH (5.3% vs. 8.1%; adjusted OR: 0.49, 95% CI: 0.11–2.19), recurrent IS (8.4% vs. 8.1%; adjusted OR: 0.96, 95% CI: 0.21–4.26), and re-occlusion during the procedure (8.4% vs. 6.3%; adjusted OR: 3.57, 95% CI: 0.90–14.29; Table 3). Figure 2 shows the distribution of the mRS scores at 90 days between the two groups. The clinical outcomes were also compared according to the timing of P2Y_12_ inhibitor administration, and no significant differences were observed (Supplementary Table 1).

**Table 1:** Baseline characteristics.

	P2Y_12_ inhibitor (+), *n* = 131	P2Y_12_ inhibitor (−), *n* = 111	*p*-value	Missing data, %
Sex, female	21 (16.0)	21 (18.9)	0.61	0
Age, years	75 (69–80)	76 (69–82)	0.46	0
Pre-stroke mRS score	0 (0–0)	0 (0–1)	0.10	0
Baseline systolic blood pressure, mmHg	163 (140–182)	156 (138–180)	0.27	2.5
Baseline NIHSS score	14 (10–19)	16 (10–22)	0.10	2.5
Medical history				
Atrial fibrillation	2 (1.5)	9 (8.1)	0.03	0
Hypertension	88 (67.2)	71 (64.0)	0.68	0
Diabetes mellitus	44 (33.6)	37 (33.3)	1.00	0
Dyslipidemia	43 (32.8)	41 (36.9)	0.59	0
Ischemic stroke/TIA prior to index stroke	15 (11.5)	20 (18.0)	0.20	0
Ischemic heart disease	15 (11.5)	14 (12.6)	0.84	0
Antithrombotic drugs prior to index stroke				
Single antiplatelet drug	22 (16.8)	22 (19.8)	0.62	0.8
Dual antiplatelet drugs	4 (3.1)	6 (5.4)	0.52	0.8
Any anticoagulant drugs	2 (1.5)	10 (9.0)	0.01	0.8
Statin	28 (21.4)	35 (31.5)	0.08	0.8
Imaging				
ASPECTS	8 (6–9)	7 (6–9)	0.87	0
c-ICA occlusion/ stenosis			1.00	0
c-ICA occlusion	77 (58.8)	66 (59.5)	–	0
c-ICA stenosis	54 (41.2)	45 (40.5)	–	0
Degree of stenosis at baseline (NASCET), % (n = 99)	100 (95–100)	100 (90–100)	0.71	0.4
Distal occluded vessel			0.45	
Intracranial internal carotid artery	41 (31.3)	31 (27.9)	–	0
M1 segment of MCA	63 (48.1)	54 (48.6)	–	0
M2 segment of MCA	27 (20.6)	26 (23.4)	–	0
Time delay				
Time from LKW to hospital arrival, min	128 (69–358)	136 (49–263)	0.25	5.8
Time from LKW to puncture, min	248 (150–445)	224 (138–371)	0.20	5.8
Time from puncture to initial mTICI ≥2a reperfusion, min	70 (48–110)	66 (40–104)	0.36	5.8
Treatment				
Intravenous thrombolysis	52 (39.7)	35 (31.5)	0.23	0
Endovascular treatment for c-ICA occlusion/ stenosis				
Stent retriever/combined contact aspiration and stent retriever	7 (5.3)	13 (11.7)	0.10	0
Contact aspiration	10 (7.6)	16 (14.4)	0.10	0
Angioplasty	53 (40.5)	59 (53.2)	0.05	0
Number of angioplasties	2 (1–2)	2 (1–2)	0.40	19.3
Carotid artery stenting	113 (86.3)	47 (42.3)	<0.01	0
Local intraarterial fibrinolysis	1 (0.8)	3 (2.7)	0.34	0
Antegrade thrombectomy	56 (42.7)	44 (39.6)	0.69	0
Antiplatelet medication during the perioperative period				
Aspirin	122 (93.1)	23 (20.7)	<0.01	0
Cilostazol	5 (3.8)	12 (10.8)	<0.01	0
Intravenous ozagrel	3 (2.3)	3 (2.7)	1.00	0

Data are presented as the median (interquartile range) or number (percentage). ASPECTS, Alberta Stroke Program Early Computed Tomography Score; c-ICA, cervical internal carotid artery; MCA, middle cerebral artery; LKW, last known well; mRS, modified Rankin Scale, NIHSS; National Institutes of Health Stroke Scale; NASCET, North America symptomatic carotid endarterectomy trial; TIA, transient ischemic attack.

**Table 2:** Procedural complications during EVT.

	P2Y_12_ inhibitor (+), *n* = 131	P2Y_12_ inhibitor (−), *n* = 111	*p*-value
Cholesterol embolus	0 (0.0)	1 (0.9)	0.46
Distal embolism	4 (3.1)	2 (1.8)	0.69
Puncture site complication	1 (0.8)	1 (0.9)	1.00
Arterial dissection	2 (1.5)	0 (0.0)	0.50
Vascular perforation	1 (0.8)	1 (0.9)	1.00
Rupture of blood vessel	0 (0.0)	1 (0.9)	0.46
Complications (intracranial)	7 (5.3)	6 (5.4)	1.00
Complications (cervical)	4 (3.1)	4 (3.6)	1.00

Data are presented as numbers (percent).

**Table 3:** Outcomes between patients with and without P2Y_12_ inhibitor.

	P2Y_12_ inhibitor (+), *n* = 131	P2Y_12_ inhibitor (−), *n* = 111	Crude OR (95% CI)	Adjusted OR (95% CI)*	Mixed effect logistic model with IPTW**
Primary outcomes					
Good functional outcome (mRS 0–2 at 90 days)	55 (42.0)	36 (32.4)	1.51 (0.89–2.56)	6.65 (1.88–23.53)	3.44 (1.03–11.43)
Secondary outcomes					
Death within 90 days	5 (3.8)	11 (9.9)	0.36 (0.12–1.07)	0.82 (0.14–4.90)	0.26 (0.06–1.12)
mRS score at 90 days	3 (2−4)	3 (2−5)	–	–	–
Any hemorrhagic event	31 (23.7)	26 (23.4)	1.01 (0.56–1.84)	0.68 (0.27–1.74)	0.28 (0.10–0.77)
Any ICH	12 (9.2)	17 (15.3)	0.56 (0.25–1.22)	0.30 (0.09–1.01)	0.14 (0.04–0.53)
Symptomatic ICH	7 (5.3)	9 (8.1)	0.64 (0.23–1.78)	0.44 (0.09–2.09)	0.20 (0.04–0.86)
Any ischemic event	11 (8.4)	5 (4.5)	1.94 (0.65–5.77)	0.93 (0.17–5.12)	2.22 (0.44–11.11)
Recurrent ischemic stroke	11 (8.4)	9 (8.1)	1.04 (0.41–2.61)	0.89 (0.20–4.09)	0.96 (0.21–4.26)
Re-occlusion after EVT	5 (3.8)	8 (7.2)	0.51 (0.16–1.61)	0.55 (0.08–4.01)	0.62 (0.15–2.58)
mRS shift (increase of 1 point)	–	–	0.70 (0.45–1.10)	0.52 (0.27–1.06)	0.76 (0.18–3.21)
Procedural outcomes					
Final mTICI ≥2b reperfusion	126 (96.2)	99 (89.2)	3.05 (1.04–8.96)	1.67 (0.31–9.03)	1.88 (0.36–9.85)
Final mTICI ≥2c reperfusion	78 (59.5)	58 (52.3)	1.34 (0.81–2.24)	1.47 (0.64–3.39)	2.49 (0.92–6.73)
Re-occlusion during procedure	11 (8.4)	7 (6.3)	1.36 (0.51–3.64)	2.78 (0.73–12.50)	2.48 (0.92–6.73)

Data are presented as median (interquartile range) or number (percent). *Adjusted for sex, age, pre-stroke mRS, baseline National Institutes of Health Stroke Scale score, hypertension, diabetes mellitus, dyslipidemia, Alberta Stroke Program Early Computed Tomography Score, intravenous thrombolysis, statin use, aspirin during the perioperative period, and angioplasty/ carotid artery stenting. **The weighted multivariable model: The model showed a c-statistics of 0.55 and a Hosmer Lemeshow chi-squared statistic of 6.00 (*p* = 0.65). CI, confidence interval; EVT, endovascular therapy; ICH, intracranial hemorrhage; IPTW, inverse probability of treatment weighting; mRS, modified Rankin Scale; mTICI, modified Thrombolysis In Cerebral Infarction scale; OR, odds ratio.

Of the total, 143 (59.1%) patients had occlusion at the c-ICA with ipsilateral intracranial artery occlusion. For both patients with occlusion and stenosis at the c-ICA with ipsilateral intracranial artery occlusion, the rate of CAS was significantly higher in the patients who received P2Y_12_ inhibitors than in those who did not receive P2Y_12_ inhibitors (Supplementary Table 2). In patients with occlusion at the c-ICA with ipsilateral intracranial artery occlusion, a significantly higher rate of good functional outcomes was found in the patients who received P2Y_12_ inhibitors than in those who did not (39.0% vs. 28.8%; adjusted 5.33, 95% CI, 1.27–22.29). However, in patients with stenosis at the c-ICA with ipsilateral intracranial artery, no significant difference was found between patients who received P2Y_12_ inhibitors in the perioperative period and those who did not (Table 4).

**Table 4:** Outcomes in the patients with occlusion or stenosis at the c-ICA with ipsilateral intracranial artery occlusion.

	P2Y_12_ inhibitor (+), *n* = 77	P2Y_12_ inhibitor (−), *n* = 66	Crude OR (95% CI)	Adjusted OR (95% CI)*
c-ICA occlusion				
Primary outcomes				
Good functional outcome (mRS 0–2 at 90 days)	30 (39.0)	19 (28.8)	1.58 (0.78–3.19)	5.33 (1.27–22.29)
Secondary outcomes				
Death within 90 days	3 (3.9)	8 (12.1)	0.29 (0.07–1.16)	0.74 (0.09–6.35)
mRS score at 90 days	3 (2−4)	4 (2−4)	–	–
Any hemorrhagic event	20 (26.0)	18 (27.2)	0.94 (0.44–1.97)	0.49 (0.15–1.57)
Any ICH	7 (9.1)	14 (21.2)	0.37 (0.14–0.99)	0.14 (0.03–0.68)
Symptomatic ICH	4 (5.2)	8 (12.1)	0.40 (0.11–1.38)	0.22 (0.03–1.55)
Any ischemic event	8 (10.4)	5 (7.6)	1.41 (0.44–4.55)	1.07 (0.17–6.64)
Recurrent ischemic stroke	6 (7.8)	6 (9.1)	0.85 (0.26–2.76)	1.01 (0.15–6.61)
Re-occlusion after EVT	2 (2.6)	5 (7.6)	0.32 (0.06–1.74)	0.28 (0.01–5.95)
mRS shift (increase of 1 point)	–	–	0.78 (0.43–1.39)	0.46 (0.18–1.14)
Procedural outcomes				
Final mTICI ≥2b reperfusion	73 (94.8)	61 (92.4)	1.50 (0.38–5.82)	0.68 (0.06–8.15)
Final mTICI ≥2c reperfusion	51 (66.2)	35 (53.0)	1.73 (0.88–3.42)	1.81 (0.59–5.56)
Re-occlusion during procedure	6 (7.8)	5 (7.6)	1.03 (0.30–3.55)	0.19 (0.03–1.13)
	P2Y_12_ inhibitor (+), *n* = 54	P2Y_12_ inhibitor (−), *n* = 45	Crude OR (95% CI)	Adjusted OR (95% CI)*
c-ICA stenosis				
Primary outcomes				
Good functional outcome (mRS 0–2 at 90 days)	25 (46.3)	17 (37.8)	1.42 (0.63–3.18)	19.65 (0.94–404.5)
Secondary outcomes				
Death within 90 days	2 (3.7)	3 (6.7)	0.29 (0.07–1.16)	–
mRS score at 90 days	3 (1−4)	3 (2−5)	–	–
Any hemorrhagic event	11 (20.4)	8 (17.8)	0.94 (0.45–1.97)	0.98 (0.16–6.02)
Any ICH	5 (9.3)	3 (6.7)	0.37 (0.14–0.99)	1.12 (0.04–28.32)
Symptomatic ICH	3 (5.6)	1 (2.2)	0.40 (0.11–1.38)	–
Any ischemic event	3 (5.6)	0 (0.0)	1.41 (0.44–4.55)	–
Recurrent ischemic stroke	5 (9.3)	3 (6.7)	0.84 (0.26–2.76)	14.69 (0.07–3,276)
Re-occlusion after EVT	3 (5.6)	3 (6.7)	0.33(0.16–1.61)	0.55 (0.08–4.01)
mRS shift (increase of 1 point)	–	–	0.78 (0.43–1.39)	0.53 (0.16–1.76)
Procedural outcomes				
Final mTICI ≥2b reperfusion	53 (98.1)	38 (84.4)	1.50 (0.38–5.82)	8.02 (0.40–160.4)
Final mTICI ≥2c reperfusion	27 (50.0)	23 (51.1)	1.74 (0.88–3.42)	1.18 (0.30–4.72)
Re-occlusion during procedure	5 (9.3)	2 (4.4)	1.03 (0.30–3.54)	1.70 (0.03–91.80)

Data are presented as median (interquartile range) or number (percent). *Adjusted for sex, age, pre-stroke mRS, baseline National Institutes of Health Stroke Scale score, hypertension, diabetes mellitus, dyslipidemia, Alberta Stroke Program Early Computed Tomography Score, intravenous thrombolysis, statin use, aspirin during the perioperative period, and angioplasty/ carotid artery stenting. CI, confidence interval; c-ICA, cervical internal carotid artery; EVT, endovascular therapy; ICH, intracranial hemorrhage; IPTW, inverse probability of treatment weighting; mRS, modified Rankin Scale; mTICI, modified Thrombolysis In Cerebral Infarction scale; OR, odds ratio.

### 3.2 Outcomes for patients receiving CAS

Of all 242 patients undergoing EVT for AIS due to extracranial carotid atherosclerosis with anterior circulation TOs, CAS was performed in 160 patients (66.1%), of whom 57/160 (35.6%) underwent angioplasty, and 103 (64.4%) underwent CAS alone. Patient background with and without carotid artery stenting is shown in Supplementary Table 3. Patients receiving P2Y_12_ inhibitors (*n* = 113) had a higher frequency of good functional outcome than those not receiving P2Y_12_ inhibitors (*n* = 47; 39.8% vs. 34.0%; adjusted OR: 4.79, 95% CI: 1.19–19.19), whereas the two groups showed no significant difference in death within 90 days (2.7% vs. 6.4%; adjusted OR: 0.16, 95% CI: 0.01–111.1) and SICH (4.4% vs. 8.5%; adjusted OR: 0.57, 95% CI: 0.06–5.34; Supplementary Table 4; Supplementary Figure 1A). Among patients who did not undergo CAS, no significant differences between patients receiving P2Y_12_ inhibitors (*n* = 18) and those not receiving P2Y_12_ inhibitors (*n* = 64) in good functional outcome (55.6% vs. 31.3%; adjusted OR: 3. 56, 95% CI: 0.74–17.13), death within 90 days (11.1% vs. 12.5%; adjusted OR: 1.13, 95% CI: 0.18–7.18) and SICH (11.1% vs. 7.8%; adjusted OR: 1.62, 95% CI: 0.26–10.04) are shown in the Supplementary Table 4 and Supplementary Figure 1B. In the overall cohort, there were no significant statistical differences between patients who underwent CAS and those who did not for good functional outcome (38.1% vs. 36.6%; adjusted OR: 0.67, 95% CI: 0.31–1.46), death within 90 days (3.8% vs. 12.2%; adjusted OR: 0.37, 95% CI: 0.10–1.43), and SICH (5.6% vs. 8.5%; adjusted OR: 0.80, 95% CI: 0.23–2.73).

### 3.3 Outcomes for receiving aspirin in the perioperative period

Among patients receiving aspirin in the perioperative period, patients receiving aspirin and P2Y_12_ inhibitors (*n* = 123) showed a higher frequency of CAS (86.9% vs. 60.9%, *p* < 0.01) and a lower frequency of cilostazol administration during the perioperative period than those receiving aspirin alone or aspirin and APT other than P2Y_12_ inhibitor (*n* = 23; 3.3% vs. 47.8%, *p* < 0.01; Supplementary Table 5). Patients receiving aspirin and P2Y_12_ inhibitors in the perioperative period had a higher frequency of good functional outcomes than those receiving aspirin alone or aspirin and APT other than P2Y_12_ inhibitor (41.0% vs. 8.7%; adjusted OR: 7.29, 95% CI: 1.64–35.48). The two groups showed no significant difference in death within 90 days (3.3% vs. 8.7%; adjusted OR: 089, 95% CI: 0.04–20.00) and SICH (4.9% vs. 13.0%; adjusted OR: 0.31, 95% CI: 0.06–1.68; Supplementary Table 6). Furthermore, the distribution of mRS scores at 90 days in patients receiving aspirin in the perioperative period in the two groups is shown in Supplementary Figure 2.

### 3.4 APT regimens for administration in the perioperative period

**Figure 3:**
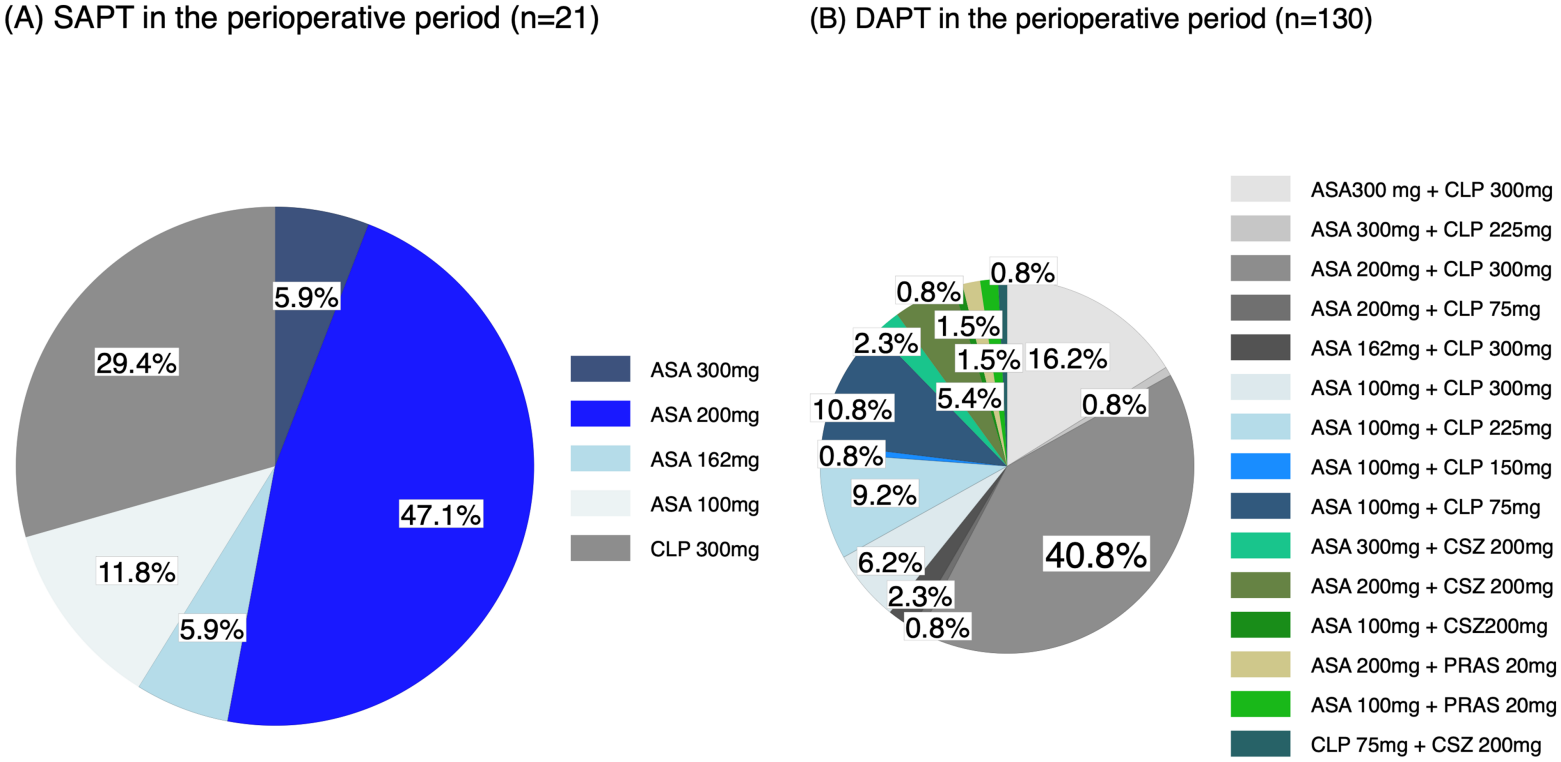
Perioperative antiplatelet drug regimens. (A) SAPT and (B) DAPT in the perioperative periods. ASA, aspirin; CLP, clopidogrel; CSZ, cilostazol; PRAS, prasugrel.

In the perioperative period, of all 242 patients, 87 (36.0%) did not receive any APT, 21 (8.7%) received SAPT, 130 (53.7%) received DAPT, which was the highest of the four groups, and 4 (1.6%) received TAPT. The most common regimen was 200 mg of aspirin (47.1%) among patients receiving SAPT. Among patients receiving DAPT, the most common regimen was 200 mg of aspirin and 300 mg of clopidogrel (40.8%), followed by 300 mg of aspirin and 300 mg of clopidogrel (16.2%; Figure 3; Supplementary Table 7). Of the four patients who received perioperative TAPT, one received 300 mg of aspirin, 30 mg of clopidogrel, and 300 mg of cilostazol; and three received 200 mg of aspirin, 30 mg of clopidogrel, and 300 mg of cilostazol. No significant difference was found in the rate of achieving a good functional outcome or death within 90 days between the perioperative antiplatelet drug regimens (Table 5). Details of the additional antithrombotic drugs administered before onset and during the perioperative period are shown in Supplementary Table 8. Furthermore, details of the pre-antithrombotic and perioperative additional antithrombotic doses are shown in Supplementary Table 9.

**Table 5:** Outcomes between patients by APT in the perioperative period.

	Not any APT, *n* = 87	SAPT, *n* = 21	DAPT*, *n* = 130	TAPT**, *n* = 4	*p*-value***
Primary outcome					
Good functional outcome (mRS 0–2 at 90 days)	34 (39.1)	7 (33.3)	48 (36.9)	2 (50.0)	0.88
Secondary outcomes					
Death within 90 days	9 (10.3)	2 (9.5)	5 (3.8)	0 (0.0)	0.21
mRS score at 90 days	3 (2−4)	4 (2−4)	3 (2−4)	3 (1−5)	0.86
Any hemorrhagic event	17 (19.5)	10 (47.6)	29 (22.3)	1 (25.0)	0.06
Any ICH	11 (12.6)	5 (23.8)	12 (9.2)	1 (25.0)	0.15
Symptomatic ICH	6 (6.9)	2 (9.5)	7 (5.4)	1 (25.0)	0.27
Any ischemic event	3 (3.4)	3 (14.3)	10 (7.7)	0 (0.0)	0.22
Recurrent ischemic stroke	7 (8.0)	3 (14.3)	10 (7.7)	0 (0.0)	0.71
Re-occlusion after procedure	7 (8.0)	1 (4.8)	5 (3.8)	0 (0.0)	0.50
Procedural outcomes					
Final mTICI ≥2c reperfusion	77 (88.5)	19 (90.5)	125 (96.2)	4 (100.0)	0.12
Final mTICI ≥2b reperfusion	46 (52.9)	13 (61.9)	75 (57.7)	2 (50.0)	0.82
Re-occlusion during procedure	3 (3.4)	1 (4.8)	14 (10.8)	0 (0.0)	0.21

Data are presented as median (interquartile range) or number (percent). *DAPT was defined as an APT with any two of aspirin, clopidogrel, or cilostazol. **TAPT was defined as APTs with any three of aspirin, clopidogrel, cilostazol, or prasugrel. ***Fisher’s exact test. APT, antiplatelet therapy; ASPECTS, Alberta Stroke Program Early Computed Tomography Score; CAS, carotid artery stenting; CI, confidence interval; DAPT, dual antiplatelet therapy; ICH, intracranial hemorrhage; mRS, modified Rankin Scale; mTICI, modified Thrombolysis In Cerebral Infarction scale; OR, odds ratio; SAPT, single antiplatelet therapy; TAPT, triple antiplatelet therapy.

## 4 Discussion

This RESCUE AT-LVO sub-study demonstrated that the perioperative administration of P2Y_12_ inhibitors during EVT for anterior circulation TO, particularly in patients with occlusion at the c-ICA with ipsilateral intracranial artery occlusion, was associated with good functional outcomes without increasing the risk of hemorrhagic complications, and the same results were observed in those who also underwent CAS or received perioperative aspirin. However, due to the small sample size and differences in patient background, caution is warranted when interpreting these results, as the correction for confounding bias was insufficient. Furthermore, perioperative antiplatelet regimens were extremely complex in actual clinical practice, and this complexity complicates the interpretation of the relationship between perioperative administration of P2Y_12_ inhibitors during EVT for anterior circulation TO and the results of the present sub-study.

The use of antiplatelet drugs in patients who have undergone CAS can reduce the incidence of intracranial embolism, carotid artery re-occlusion, and stent thrombosis (5); however, data to support these practices are lacking. Considering the strong evidence supporting P2Y_12_ inhibitors combined with aspirin for reducing stent thrombosis risk after coronary stenting, the administration of intraoperative P2Y_12_ inhibitors to patients with AIS due to anterior circulation TOs, particularly those undergoing CAS (39) might have contributed to improved outcomes by lowering the risk of post-CAS stent thrombosis (40). Pop et al. evaluated the predictors of delayed stent thrombosis in 81 patients with TOs undergoing CAS and found that the rate of stent occlusion was significantly lower in patients treated with aspirin and clopidogrel than in those treated with aspirin alone (41). Our study showed no significant difference in the incidence of post-procedure re-occlusion in relation to the number of antiplatelet agents administered during EVT. In the present sub-study, patients who received P2Y_12_ inhibitors had significantly more CAS procedures than those who did not receive P2Y_12_ inhibitors. This finding was believed to be because P2Y_12_ inhibitors, which are antiplatelet agents, were usually used perioperatively in patients undergoing CAS to prevent intravascular thrombosis caused by platelet activation due to intimal injury or stent placement. Based on previous studies showing that patients who underwent angioplasty and CAS had a better outcome than those who underwent angioplasty alone in patients with TOs (38), although we performed IPTW in addition to multivariate analysis, our results could not completely exclude the possibility that CAS influenced the achievement of good functional outcomes due to various confounding biases.

Moreover, in our results, patients receiving P2Y_12_ inhibitors received significantly less cilostazol perioperatively than those not receiving P2Y_12_ inhibitors, while the rate of aspirin administration was significantly higher in patients receiving P2Y_12_ inhibitors. Cilostazol, a phosphodiesterase III inhibitor, was selected for perioperative antiplatelet therapy over P2Y_12_ inhibitors because it is as effective as aspirin in the treatment of acute non-cardioembolic stroke (42), as well as the expected benefit of reducing the risk of bleeding complication (43) and promoting atherosclerotic plaque regression (44). Additionally, the physicians might have opted for cilostazol instead of a P2Y_12_ inhibitor in the perioperative period for patients in who are unable to take P2Y_12_ inhibitors for some reason.

In our results, a large number of drug combinations were used for perioperative APT, including SAPT, DAPT, and TAPT; although aspirin or clopidogrel was primarily used, different types of antiplatelet drugs were also used, which was one of the factors that made interpreting the analysis results was difficult. Several perioperative APT regimens have been proposed for patients undergoing CAS for stenosis or occlusive lesions, including regimens without APT, monotherapy with aspirin alone, which resulted in a stent occlusion rate of 10.3% within 7 days, and DAPT with a combination of aspirin and clopidogrel (45). DAPT is the most commonly used APT regimens in many previous studies and provides a marginal benefit in terms of good functional outcomes for CAS without significantly increasing the risk of SICH (45). Previous studies in acute TO have reported the use of an intravenous loading dose of aspirin (250–500 mg) and an oral intake of a loading dose of clopidogrel (300 mg), which can be administered immediately without prior intravenous thrombolysis (14, 45) or 24 h later after the exclusion of bleeding on postprocedural follow-up CT (46), followed by DAPT for 3 months. In other studies, patients received aspirin (100 mg) and clopidogrel (75 mg) 24 h after CAS placement (47). As reported in previous studies, oral aspirin has long been used by physicians for platelet suppression after elective EVT (48). The complexities of perioperative APT regimens noted in the Thrombectomy In TANdem Occlusion (TITAN) registry include intravenous aspirin, intravenous GP IIb/IIIa receptor antagonist, clopidogrel, and unfractionated heparin (49). Currently, several RCTs are underway to verify the effectiveness of EVT for TO, and each RCT has a standardized perioperative APT regimen. Regarding perioperative APT, the protocol of the Endovascular Acute Stroke Intervention-Tandem OCclusion Trial (NCT04261478) states that (1) in patients who have not been treated with intravenous thrombolysis, oral or intrarectal SAPT (325 mg of aspirin orally or 650 mg by rectum) is given immediately after the procedure, and a second agent (usually clopidogrel 300 mg orally) is added after follow-up brain imaging at 12–24 h confirms the absence of significant ICH; (2) For patients not treated with intravenous thrombolysis, DAPT (325 mg of aspirin orally or 650 mg rectally and 300–600 mg of clopidogrel orally) is given immediately after the procedure; and (3) routine use of GP IIb/IIIa inhibitors, periprocedural intravenous heparin is discouraged. Additionally, the protocol of TITAN (NCT03978988) (50) states (1) the use of intravenous aspirin (250 mg); (2) a DAPT is administered after 24 (6) h of imaging follow-up excluding ICH; and (3) the type and dose of DAPT is left to the discretion of the local practice. Therefore, to determine the optimal perioperative APT for patients with TOs during the perioperative period in the future, it will be necessary to compare them using a standardized regimen.

Limitations of the present sub-study include its retrospective analysis, non-randomized design, and heterogeneous antithrombotic protocols. Therefore, in the patients performed CAS, the observed benefit of P2Y_12_ inhibitors in the perioperative period may have been secondary to improved recanalization with CAS. Second, the association between P2Y_12_ inhibitors and clinical outcome was statistically significantly different, with the addition of intraoperative aspirin as an adjustment factor suggesting that its addition may conflict with the issue of multicollinearity. Third, cangrelor and/or tirofiban were not used in this study because they are not approved for use in Japan. Cangrelor, a P2Y_12_ inhibitor and an active drug that does not require metabolic conversion, has been reported to be effective in recent studies and showed a safety profile similar to the commonly used DAPT loading protocols in patients with acute tandem lesions in an international multicenter cohort (51). Tirofiban, a GP IIb/IIIa inhibitor, improved functional outcomes independent of premedication in patients with stroke due to acute extracranial carotid lesions and emergency CAS with lower rates of SICH (52). Finally, despite its multicenter design, our study may have been underpowered to detect differences in outcomes between the two groups.

In conclusion, the perioperative administration of P2Y_12_ inhibitors might be associated with a higher frequency of good functional outcomes in patients undergoing EVT for AIS with anterior circulation TOs. However, this sub-analysis of EVT for anterior circulation TOs included several confounding factors; therefore, further studies are warranted.

## Glossary

**Table:** 

TOs	Tandem Occlusions
c-ICA	Cervical Internal Carotid Artery
ICA	Internal Carotid Artery
CT	Computed Tomography
MCA	Middle Cerebral Artery
IS	Ischemic Stroke
MR	Magnetic Resonance
ASPECTS	Alberta Stroke Program Early Computed Tomographic Score
AIS	Acute Ischemic Stroke
ORs	Odds Ratios
CIs	Confidence Intervals
RCTs	Randomized Controlled Trials
CAS	Carotid Artery Stenting
NIHSS	National Institutes of Health Stroke Scale
mTICI	Modified Thrombolysis In Cerebral Infarction Scale
EVT	Endovascular Therapy
IPTW	Inverse Probability of Treatment Weighting
TITAN	Thrombectomy In TANdem OCclusion
IQR	Interquartile Range
mRS	Modified Rankin Scale
APT	Antiplatelet Therapy
SAPT	Single Antiplatelet Therapy
DAPT	Dual Antiplatelet Therapy
SICH	Symptomatic Intracranial Hemorrhage
ICH	Intracranial Hemorrhage
LKW	Last Known Well
LVO	Large Vessel Occlusion
RESCUE AT-LVO	Recovery by Endovascular Salvage for Cerebral Ultra-acute Embolic and Atherothrombotic Stroke with Large Vessel Occlusion
GP	Glycoprotein
TAPT	Triple Antiplatelet Therapy

## Data Availability

The original contributions presented in the study are included in the article/Supplementary material, further inquiries can be directed to the corresponding author.

## References

[1] Jadhav AP Zaidat OO Liebeskind DS Yavagal DR Haussen DC Hellinger FR Jr. Emergent management of tandem lesions in acute ischemic stroke. Stroke. (2019) 50:428–33. doi: 10.1161/STROKEAHA.118.021893, PMID: 30580729

[2] Jacquin G Poppe AY Labrie M Daneault N Deschaintre Y Gioia LC. Lack of consensus among stroke experts on the optimal management of patients with acute tandem occlusion. Stroke. (2019) 50:1254–6. doi: 10.1161/STROKEAHA.118.023758, PMID: 30890115

[3] Saver JL Goyal M Bonafe A Diener HC Levy EI Pereira VM. Stent-retriever thrombectomy after intravenous t-PA vs. t-PA alone in stroke. N Engl J Med. (2015) 372:2285–95. doi: 10.1056/NEJMoa1415061, PMID: 25882376

[4] Campbell BCV Mitchell PJ Kleinig TJ Dewey HM Churilov L Yassi N. Endovascular therapy for ischemic stroke with perfusion-imaging selection. N Engl J Med. (2015) 372:1009–18. doi: 10.1056/NEJMoa1414792 25671797

[5] Bracard S Ducrocq X Mas JL Soudant M Oppenheim C Moulin T. Mechanical thrombectomy after intravenous alteplase versus alteplase alone after stroke (THRACE): a randomised controlled trial. Lancet Neurol. (2016) 15:1138–47. doi: 10.1016/S1474-4422(16)30177-6, PMID: 27567239

[6] Poppe AY Jacquin G Roy D Stapf C Derex L. Tandem carotid lesions in acute ischemic stroke: mechanisms, therapeutic challenges, and future directions. AJNR Am J Neuroradiol. (2020) 41:1142–8. doi: 10.3174/ajnr.A6582, PMID: 32499251 PMC7357657

[7] Powers WJ Rabinstein AA Ackerson T Adeoye OM Bambakidis NC Becker K. Guidelines for the early management of patients with acute ischemic stroke: 2019 update to the 2018 guidelines for the early management of acute ischemic stroke: a guideline for healthcare professionals from the American Heart Association/American Stroke Association. Stroke. (2019) 50:e344–418. doi: 10.1161/STR.0000000000000211, PMID: 31662037

[8] Bonati LH Kakkos S Berkefeld J de Borst GJ Bulbulia R Halliday A. European stroke organisation guideline on endarterectomy and stenting for carotid artery stenosis. Eur Stroke J. (2021) 6:I–XLVII. doi: 10.1177/23969873211012121, PMID: 34414302 PMC8370069

[9] Dalainas I Nano G Bianchi P Stegher S Malacrida G Tealdi DG. Dual antiplatelet regime versus acetyl-acetic acid for carotid artery stenting. Cardiovasc Radiol. (2006) 29:519–21. doi: 10.1007/s00270-005-5288-y, PMID: 16565792

[10] Paciaroni M Bogousslavsky J. Antithrombotic therapy in carotid artery stenosis: an update. Eur Neurol. (2015) 73:51–6. doi: 10.1159/000367988, PMID: 25402664

[11] Chaturvedi S Yadav JS. The role of antiplatelet therapy in carotid stenting for ischemic stroke prevention. Stroke. (2006) 37:1572–7. doi: 10.1161/01.STR.0000221298.43117.be, PMID: 16627791

[12] Zhu F Anadani M Labreuche J Spiotta A Turjman F Piotin M. Impact of antiplatelet therapy during endovascular therapy for tandem occlusions: a collaborative pooled analysis. Stroke. (2020) 51:1522–9. doi: 10.1161/STROKEAHA.119.028231, PMID: 32188367

[13] Juan J C Celina C Matias L Esteban V S Javier L Ivan L. Endovascular management of tandem occlusions in stroke: treatment strategies in a real-world scenario. J Neurosci Neurol Disord. (2021) 5:055–60. doi: 10.29328/journal.jnnd.1001051

[14] Diana F Abdalkader M Behme D Li W Maurer CJ Pop R. Antithrombotic regimen in emergent carotid stenting for acute ischemic stroke due to tandem occlusion: a meta-analysis of aggregate data. J Neurointerv Surg. (2024) 16:243–7. doi: 10.1136/jnis-2023-020204, PMID: 37185107

[15] Aggarwal D Bhatia K Chunawala ZS Furtado RHM Mukherjee D Dixon SR. P2Y_12_ inhibitor versus aspirin monotherapy for secondary prevention of cardiovascular events: meta-analysis of randomized trials. Eur Heart J Open. (2022) 2:oeac019. doi: 10.1093/ehjopen/oeac019, PMID: 35919116 PMC9242055

[16] Goyal M Yoshimura S Milot G Fiehler J Jayaraman M Dorn F. Considerations for antiplatelet management of carotid stenting in the setting of mechanical thrombectomy: a Delphi consensus statement. AJNR Am J Neuroradiol. (2020) 41:2274–9. doi: 10.3174/ajnr.A6888, PMID: 33122218 PMC7963237

[17] Von Elm E Altman DG Egger M Pocock SJ Gøtzsche PC Vandenbroucke JP. The strengthening the reporting of observational studies in epidemiology (STROBE) statement: guidelines for reporting observational studies. Ann Intern Med. (2007) 147:573–7. doi: 10.7326/0003-4819-147-8-200710160-00010 17938396

[18] Uchida K Yamagami H Sakai N Shirakawa M Beppu M Toyoda K. Endovascular therapy for acute intracranial large vessel occlusion due to atherothrombosis: multicenter historical registry. J Neurointerv Surg. (2024) 16:884–91. doi: 10.1136/jnis-2023-020670, PMID: 37648433

[19] Beppu M Uchida K Sakai N Yamagami H Toyoda K Matsumaru Y. Optimal endovascular therapy technique for isolated intracranial Atherothrombotic stroke-related large-vessel occlusion in the acute-to-subacute stage. AJNR Am J Neuroradiol. (2024):ajnr.A8399. doi: 10.3174/ajnr.A8399, PMID: 38951032 PMC11543085

[20] Inzitari D Eliasziw M Gates P Sharpe BL Chan RK Meldrum HE. The causes and Risk of stroke in patients with asymptomatic internal-carotid-artery stenosis. N Engl J Med. (2000) 342:1693–701. doi: 10.1056/NEJM200006083422302 10841871

[21] Toyoda K Koga M Iguchi Y Itabashi R Inoue M Okada Y. Guidelines for intravenous thrombolysis (recombinant tissue-type plasminogen activator), the third edition, march 2019: a guideline from the Japan stroke society. Neurol Med Chir (Tokyo). (2019) 59:449–91. doi: 10.2176/nmc.st.2019-0177, PMID: 31801934 PMC6923159

[22] Yamagami H Hayakawa M Inoue M Iihara K Ogasawara K Toyoda K. Guidelines for mechanical Thrombectomy in Japan, the fourth edition, march 2020: a guideline from the Japan stroke society, the Japan neurosurgical society, and the Japanese Society for Neuroendovascular Therapy. Neurol Med Chir (Tokyo). (2021) 61:163–92. doi: 10.2176/nmc.nmc.st.2020-0357, PMID: 33583863 PMC7966209

[23] Lapergue B Labreuche J Blanc R Marnat G Consoli A Rodesch G. Combined use of contact aspiration and the stent retriever technique versus stent retriever alone for recanalization in acute cerebral infarction: the randomized ASTER 2 study protocol. J Neurointerv Surg. (2020) 12:471–6. doi: 10.1136/neurintsurg-2019-014735, PMID: 31915208

[24] Almekhlafi MA Mishra S Desai JA Nambiar V Volny O Goel A. Not all "successful" angiographic reperfusion patients are an equal validation of a modified TICI scoring system. Interv Neuroradiol. (2014) 20:21–7. doi: 10.15274/INR-2014-10004 24556296 PMC3971136

[25] Kitazono T Kamouchi M Matsumaru Y Shirai T Takita A Kuroda T. Comparison of prasugrel and clopidogrel in thrombotic stroke patients with risk factors for ischemic stroke recurrence: an integrated analysis of PRASTRO-I, PRASTRO-II, and PRASTRO-III. Cerebrovasc Dis. (2023) 52:720–9. doi: 10.1159/000529149, PMID: 37011599

[26] von Kummer R Broderick JP Campbell BCV Demchuk A Goyal M Hill MD. The Heidelberg bleeding classification. Stroke. (2015) 46:2981–6. doi: 10.1161/STROKEAHA.115.010049 26330447

[27] Uchida K Yoshimura S Sakai N Yamagami H Morimoto T RESCUE-Japan Registry 2 Investigators. Sex differences in management and outcomes of acute ischemic stroke with large vessel occlusion. Stroke. (2019) 50:1915–8. doi: 10.1161/STROKEAHA.119.025344, PMID: 31167622

[28] Goyal M Menon BK van Zwam WH Dippel DWJ Mitchell PJ Demchuk AM. Endovascular thrombectomy after large-vessel ischaemic stroke: a meta-analysis of individual patient data from five randomised trials. Lancet. (2016) 387:1723–31. doi: 10.1016/S0140-6736(16)00163-X, PMID: 26898852

[29] Gilgen MD Klimek D Liesirova KT Meisterernst J Klinger-Gratz PP Schroth G. Younger stroke patients with large pretreatment diffusion-weighted imaging lesions may benefit from endovascular treatment. Stroke. (2015) 46:2510–6. doi: 10.1161/STROKEAHA.115.010250, PMID: 26251252

[30] Gautheron V Xie Y Tisserand M Raoult H Soize S Naggara O. Outcome after reperfusion therapies in patients with large baseline diffusion-weighted imaging stroke lesions: a THRACE trial (mechanical thrombectomy after intravenous alteplase versus alteplase alone after stroke) subgroup analysis. Stroke. (2018) 49:750–3. doi: 10.1161/STROKEAHA.117.020244, PMID: 29382803

[31] Kobeissi H Ghozy S Seymour T Gupta R Bilgin C Kadirvel R. Outcomes of patients with atrial fibrillation following thrombectomy for stroke: a systematic review and meta-analysis. JAMA Netw Open. (2023) 6:e2249993. doi: 10.1001/jamanetworkopen.2022.49993, PMID: 36607633 PMC9857225

[32] Flint AC Conell C Ren X Banki NM Chan SL Rao VA. Effect of systolic and diastolic blood pressure on cardiovascular outcomes. N Engl J Med. (2019) 381:243–51. doi: 10.1056/NEJMoa1803180 31314968

[33] Sarwar N Gao P Seshasai SR Gobin R Kaptoge S di Angelantonio E. Diabetes mellitus, fasting blood glucose concentration, and risk of vascular disease: a collaborative meta-analysis of 102 prospective studies. Lancet. (2010) 375:2215–22. doi: 10.1016/S0140-6736(10)60484-9, PMID: 20609967 PMC2904878

[34] Iso H Jacobs DR Wentworth D Neaton JD Cohen JD for the MRFIT Research Group*. Serum cholesterol levels and six-year mortality from stroke in 350,977 men screened for the multiple risk factor intervention trial. N Engl J Med. (1989) 320:904–10. doi: 10.1056/NEJM198904063201405, PMID: 2619783

[35] Yoshimoto T Inoue M Yamagami H Fujita K Tanaka K Ando D. Use of diffusion-weighted imaging-Alberta stroke program early computed tomography score (DWI-ASPECTS) and ischemic core volume to determine the malignant profile in acute stroke. J Am Heart Assoc. (2019) 8:e012558. doi: 10.1161/JAHA.119.012558, PMID: 31698986 PMC6915267

[36] Baigent C Blackwell L Emberson J Holland LE Reith C Bhala N. Efficacy and safety of more intensive lowering of LDL cholesterol: a meta-analysis of data from 170,000 participants in 26 randomised trials. Lancet. (2010) 376:1670–81. doi: 10.1016/S0140-6736(10)61350-5, PMID: 21067804 PMC2988224

[37] Chen ZM Sandercock P Pan HC Counsell C Collins R Liu LS. Indications for early aspirin use in acute ischemic stroke: a combined analysis of 40 000 randomized patients from the chinese acute stroke trial and the international stroke trial. On behalf of the CAST and IST collaborative groups. Stroke. (2000) 31:1240–9. doi: 10.1161/01.STR.31.6.1240, PMID: 10835439

[38] Zevallos CB Farooqui M Quispe-Orozco D Mendez-Ruiz A Dajles A Garg A. Acute carotid artery stenting versus balloon angioplasty for tandem occlusions: a systematic review and meta-analysis. J Am Heart Assoc. (2022) 11:e022335. doi: 10.1161/JAHA.121.022335, PMID: 35023353 PMC9238531

[39] Virk HUH Escobar J Rodriguez M Bates ER Khalid U Jneid H. Dual antiplatelet therapy: a concise review for clinicians. Life (Basel). (2023) 13:1580. doi: 10.3390/life13071580, PMID: 37511955 PMC10381391

[40] Aradi D Kirtane A Bonello L Gurbel PA Tantry US Huber K. Bleeding and stent thrombosis on P2Y_12_-inhibitors: collaborative analysis on the role of platelet reactivity for risk stratification after percutaneous coronary intervention. Eur Heart J. (2015) 36:1762–71. doi: 10.1093/eurheartj/ehv104, PMID: 25896078

[41] Pop R Zinchenko I Quenardelle V Mihoc D Manisor M Richter JS. Predictors and clinical impact of delayed stent thrombosis after thrombectomy for acute stroke with tandem lesions. AJNR Am J Neuroradiol. (2019) 40:533–9. doi: 10.3174/ajnr.A5976, PMID: 30765378 PMC7028646

[42] Lee YK Bae HJ Kang DW Lee SH Yu K Park JM. Cilostazol in acute ischemic stroke treatment (CAIST trial): a randomized double-blind non-inferiority trial. Cerebrovasc Dis. (2011) 32:65–71. doi: 10.1159/000327036, PMID: 21613787

[43] Hoshino H Toyoda K Omae K Ishida N Uchiyama S Kimura K. Dual antiplatelet therapy using cilostazol with aspirin or clopidogrel: subanalysis of the CSPS.com trial. Stroke. (2021) 52:3430–9. doi: 10.1161/STROKEAHA.121.034378, PMID: 34404237 PMC8547582

[44] Sohn M Chun EJ Lim S. Cilostazol treatment for preventing adverse cardiovascular events in patients with type 2 diabetes and coronary atherosclerosis: long-term follow-up of the ESCAPE study. J Diabetes. (2022) 14:524–31. doi: 10.1111/1753-0407.13300, PMID: 35932165 PMC9426278

[45] Hellegering J Uyttenboogaart M Bokkers RPH Moumni ME Zeebregts CJ van der Laan MJ. Treatment of the extracranial carotid artery in tandem lesions during endovascular treatment of acute ischemic stroke: a systematic review and meta-analysis. Ann Transl Med. (2020) 8:1278. doi: 10.21037/atm-2020-cass-17, PMID: 33178810 PMC7607118

[46] Neuberger U Moteva K Vollherbst DF Schönenberger S Reiff T Ringleb PA. Tandem occlusions in acute ischemic stroke -impact of antithrombotic medication and complementary heparin on clinical outcome and stent patency. J Neurointerv Surg. (2020) 12:1088–93. doi: 10.1136/neurintsurg-2019-015596, PMID: 31937604

[47] Marnat G Mourand I Eker O Machi P Arquizan C Riquelme C. Endovascular management of tandem occlusion stroke related to internal carotid artery dissection using a distal to proximal approach: insight from the RECOST study. AJNR Am J Neuroradiol. (2016) 37:1281–8. doi: 10.3174/ajnr.A4752, PMID: 26965467 PMC7960330

[48] Li W Chen Z Dai Z Liu R Yin Q Wang H. Management of acute tandem occlusions: stent-retriever thrombectomy with emergency stenting or angioplasty. J Int Med Res. (2018) 46:2578–86. doi: 10.1177/0300060518765310, PMID: 29726291 PMC6124263

[49] Anadani M Spiotta AM Alawieh A Turjman F Piotin M Haussen DC. Emergent carotid stenting plus Thrombectomy after thrombolysis in tandem strokes: analysis of the TITAN registry. Stroke. (2019) 50:2250–2. doi: 10.1161/STROKEAHA.118.024733, PMID: 31577899

[50] Zhu F Hossu G Soudant M Richard H Achit H Beguinet M. Effect of emergent carotid stenting during endovascular therapy for acute anterior circulation stroke patients with tandem occlusion: a multicenter, randomized, clinical trial (TITAN) protocol. Int J Stroke. (2021) 16:342–8. doi: 10.1177/1747493020929948, PMID: 32515696

[51] Rodriguez-Calienes A Oliver M Hassan AE Vivanco-Suarez J Divani AA Ribo M. Safety of intravenous cangrelor versus dual oral antiplatelet loading therapy in endovascular treatment of tandem lesions: An observational cohort study. Stroke: Vasc Intervent Neurol. (2023) 3:e001020. doi: 10.1161/SVIN.123.001020 PMC12778733 41585048

[52] Garayzade R Berlis A Schiele S Ertl M Schneider H Müller G. Efficacy and safety outcomes for acute ischemic stroke patients treated with intravenous infusion of tirofiban after emergent carotid artery stenting. Clin Neuroradiol. (2024) 34:163–72. doi: 10.1007/s00062-023-01350-7, PMID: 37796321 PMC10881598

