# Supplementary material for: Association of perioperative P2Y12 inhibitor administration with outcomes for tandem occlusion: RESCUE AT-LVO sub-study
Source: Front Neurol. 2024 Nov 21;15:1475882. doi: 10.3389/fneur.2024.1475882 (PMC11617547; doi:10.3389/fneur.2024.1475882)
Supplement: Supplementary file 1 [file Data_Sheet_1.pdf]

Supplementary Material

Association of Perioperative P2Y12 Inhibitor Administration with Outcomes for Tandem Occlusion: RESCUE AT-LVO Sub-study

Authors:

Takeshi Yoshimoto, MD, PhD[1,2]; Hiroshi Yamagami, MD, PhD[1,3,4]; Nobuyuki Sakai, MD, PhD[5]; Kazutaka Uchida, MD, PhD[6]; Manabu Shirakawa, MD, PhD[6]; Mikiya Beppu, MD, PhD[6]; Kazunori Toyoda, MD, PhD[7]; Yuji Matsumaru, MD, PhD[1,8]; Yasushi Matsumoto, MD[9]; Kenichi Todo, MD, PhD[10]; Mikito Hayakawa, MD, PhD[1,11]; Seigo Shindo, MD, PhD[12]; Masafumi Morimoto, MD[13]; Masataka Takeuchi, MD, PhD[14]; Hirotoshi Imamura, MD, PhD[15]; Hiroyuki Ikeda, MD, PhD[16]; Kanta Tanaka, MD, PhD[7,17]; Hideyuki Ishihara, MD, PhD[18]; Hiroto Kakita, MD, PhD[6]; Takanori Sano, MD, PhD[19,20]; Hayato Araki, MD, PhD[21]; Tatsufumi Nomura, MD, PhD[22]; Fumihiro Sakakibara, MD, PhD[6]; Shinichi Yoshimura MD, PhD[6]; RESCUE AT-LVO Investigators

Affiliations:

1. Department of Stroke and Cerebrovascular Diseases, University of Tsukuba Hospital, Tsukuba, Japan
2. Department of Neurology, National Cerebral and Cardiovascular Center, Suita, Japan
3. Division of Stroke Prevention and Treatment, Institute of Medicine, University of Tsukuba, Tsukuba, Japan
4. Department of Stroke Neurology, NHO Osaka National Hospital, Osaka, Japan
5. Department of Neurosurgery, Seijinkai Shimizu Hospital, Kyoto, Japan
6. Department of Neurosurgery, Hyogo Medical University, Nishinomiya, Japan
7. Department of Cerebrovascular Medicine, National Cerebral and Cardiovascular Center, Suita, Japan
8. Department of Neurosurgery, Institute of Medicine, University of Tsukuba, Tsukuba, Japan
9. Division of Development and Discovery of Interventional Therapy, Tohoku University Hospital, Sendai, Japan
10. Stroke Center, Osaka University Graduate School of Medicine, Suita, Japan
11. Department of Neurology, Institute of Medicine, University of Tsukuba, Tsukuba, Japan
12. Department of Neurology, Japanese Red Cross Kumamoto Hospital, Kumamoto, Japan
13. Department of Neurosurgery, Yokohama Shintoshi Neurosurgical Hospital, Yokohama, Japan
14. Department of Neurosurgery, Seisho Hospital, Odawara, Japan
15. Department of Neurosurgery, National Cerebral and Cardiovascular Center, Suita, Japan

16. Department of Neurosurgery, Kurashiki Central Hospital, Kurashiki, Japan
17. Stroke Center, Kindai University Hospital, Sayama, Japan
18. Department of Neurosurgery, Yamaguchi University School of Medicine, Ube, Japan
19. Department of Neurosurgery, Japanese Red Cross Ise Hospital, Ise, Japan
20. Department of Neurosurgery, Mie Prefectural General Medical Center, Yokkaichi, Japan
21. Department of Neurosurgery, Araki Neurosurgical Hospital, Hiroshima, Japan
22. Department of Neurosurgery, Ohkawara Neurosurgical Hospital, Muroran, Japan

1 Supplementary Tables

Supplementary Table 1. Outcomes by the timing of $P2Y_{12}$ inhibitor administration

	No $P2Y_{12}$ inhibitor, n=111	$P2Y_{12}$ inhibitor immediately before EVT, n=31	$P2Y_{12}$ inhibitor during EVT, n=81	$P2Y_{12}$ inhibitor immediately after EVT, n=19	P-value
**Primary outcomes**					
Good functional outcome (mRS 0–2 at 90 days)	36 (32.4)	17 (54.8)	29 (35.8)	9 (47.4)	0.11
**Secondary outcomes**					
Favorable outcome (mRS 0–3 at 90 days)	56 (50.5)	22 (71.0)	42 (51.9)	11 (57.9)	0.22
Death within 90 days	11 (9.9)	1 (3.2)	4 (4.9)	0 (0.0)	0.36
mRS score at 90 days	3 (2–5)	2 (2–4)	3 (2–4)	3 (1–4)	0.13
Any hemorrhagic event	26 (23.4)	5 (16.1)	21 (25.9)	5 (26.3)	0.72
Any ICH	17 (15.3)	4 (12.9)	8 (9.9)	0 (0.0)	0.26
Symptomatic ICH	9 (8.1)	3 (9.7)	4 (4.9)	0 (0.0)	0.53
Any ischemic event	5 (4.5)	1 (3.2)	9 (11.1)	1 (5.3)	0.29
Recurrent ischemic stroke	9 (8.1)	0 (0.0)	10 (12.3)	1 (5.3)	0.18
Re-occlusion after EVT	8 (7.2)	0 (0.0)	4 (4.9)	1 (5.3)	0.50
**Procedural outcomes**					
Final mTICI ≥ 2c reperfusion	99 (89.2)	31 (100.0)	78 (96.3)	17 (89.5)	0.07
Final mTICI ≥ 2b reperfusion	58 (52.3)	23 (74.2)	45 (55.6)	10 (52.6)	0.18
Re-occlusion during procedure	7 (6.3)	2 (6.5)	9 (11.1)	0 (0.0)	0.44

Data are presented as median (interquartile range) or number (percent).
CI, confidence interval; EVT, endovascular therapy; ICH, intracranial hemorrhage; IPTW, inverse probability of treatment weighting; mRS, modified Rankin Scale; mTICI, modified Thrombolysis In Cerebral Infarction scale; OR, odds ratio.

Supplementary Table 2. Patient background with occlusion or stenosis at the c-ICA with ipsilateral intracranial artery occlusion

	Occlusion, n=143			Stenosis, n=99		
	**$P2Y_{12}$ inhibitor (+), n=77**	**$P2Y_{12}$ inhibitor (-), n=66**	**P-value**	**$P2Y_{12}$ inhibitor (+), n=54**	**$P2Y_{12}$ inhibitor (-), n=45**	**P-value**
Sex, female	12 (15.6)	11 (16.7)	1.00	9 (16.7)	10 (22.2)	0.61
Age, years	76 (69─80)	74 (68─81)	0.44	73 (69─79)	78 (72─82)	0.03
Prestroke mRS score	0 (0─0)	0 (0─0)	0.18	0 (0─0)	0 (0─1)	0.33
Baseline systolic blood pressure, mmHg	163 (139─182)	156 (137─181)	0.48	163 (147─181)	159 (141─177)	0.41
Baseline NIHSS score	14 (12─19)	16 (12─22)	0.32	13 (8─21)	16 (9─22)	0.15
**Medical history**						
Atrial fibrillation	1 (1.3)	4 (6.1)	0.18	1 (1.9)	5 (11.1)	0.09
Hypertension	52 (67.5)	40 (60.6)	0.48	36 (66.7)	31 (68.9)	0.83
Diabetes mellitus	28 (36.4)	20 (30.3)	0.48	16 (29.6)	17 (37.8)	0.40
Dyslipidemia	24 (31.2)	22 (33.3)	0.86	19 (35.2)	19 (42.2)	0.54
Ischemic stroke/TIA prior to index stroke	10 (13.0)	12 (18.2)	0.49	5 (9.3)	8 (17.8)	0.24
Ischemic heart disease	10 (13.0)	8 (12.1)	1.00	5 (9.3)	6 (13.3)	0.54
**Antithrombotic drugs prior to index stroke**						
Single antiplatelet drug	15 (19.5)	13 (19.7)	1.00	7 (13.0)	9 (20.0)	0.42
Dual antiplatelet drugs	2 (2.6)	4 (6.1)	0.41	2 (3.7)	2 (4.4)	1.00
Statin	16 (20.8)	17 (25.6)	0.55	12 (22.2)	18 (40.0)	0.08
**Imaging**						
ASPECTS	7 (6─9)	8 (6─9)	0.67	8 (6─9)	7 (5─9)	0.83
**Distal occluded vessel**			0.82			0.13
Intracranial internal carotid artery	24 (31.2)	18 (27.3)	--	17 (31.5)	13 (28.9)	--
M1 segment of MCA	42 (54.5)	35 (53.0)	--	21 (38.9)	19 (42.2)	--
M2 segment of MCA	11 (14.3)	13 (19.7)	--	16 (29.6)	13 (28.9)	--
**Time delay**						
Time from LKW to hospital arrival, min	118 (69─300)	116 (48─218)	0.21	140 (60─417)	194.00 (50─428)	0.66

Time from LKW to puncture, min	202 (145–384)	215 (141–285)	0.68	310 (165–665)	250 (138–504)	0.18
Time from puncture to first mTICI ≥ 2a reperfusion, min	60 (42–105)	77 (47–106)	0.37	76 (52–110)	48 (36–82)	<0.01
**Treatment**						
Intravenous thrombolysis	28 (36.3)	24 (36.4)	1.00	24 (44.4)	11 (24.4)	0.06
**Endovascular therapy**						
Stent retriever/combined contact aspiration and stent retriever	4 (5.2)	10 (15.2)	0.05	3 (5.6)	3 (6.7)	1.00
Contact aspiration	8 (10.4)	13 (19.7)	0.16	2 (3.7)	3 (6.7)	0.66
Angioplasty	34 (44.2)	44 (66.7)	<0.01	19 (35.2)	15 (33.3)	1.00
Carotid artery stenting	71 (92.2)	33 (50.0)	<0.01	42 (77.8)	14 (31.1)	<0.01
Local intraarterial fibrinolysis	1 (1.3)	1 (1.5)	1.00	0 (0.0)	2 (4.4)	0.20
Antegrade thrombectomy	1 (1.3)	1 (1.5)	1.00	22 (40.7)	27 (60.0)	0.07
**Antiplatelet medication in the perioperative period**						
Aspirin	70 (90.9)	13 (19.7)	<0.01	52 (96.3)	10 (22.2)	<0.01
Cilostazol	3 (3.9)	7 (10.6)	0.19	2 (3.7)	5 (11.1)	0.24
Intravenous ozagrel	2 (2.6)	1 (1.5)	1.00	1 (1.9)	2 (4.4)	0.59

Data are presented as the median (interquartile range) or number (percent).

ASPECTS indicates Alberta Stroke Program Early Computed Tomography Score; c-ICA, cervical internal carotid artery; LKW, last known well; MCA, middle cerebral artery; mRS, modified Rankin Scale; mTICI, modified Thrombolysis In Cerebral Infarction scale; NASCET, North America symptomatic carotid endarterectomy trial; NIHSS, National Institutes of Health Stroke Scale; TIA, transient ischemic attack.

Supplementary Table 3. Patient background with and without carotid artery stenting

	CAS (+), n=160			CAS (-), n=82		
	**$P2Y_{12}$ inhibitor (+), n=113**	**$P2Y_{12}$ inhibitor (-), n=47**	**P-value**	**$P2Y_{12}$ inhibitor (+), n=18**	**$P2Y_{12}$ inhibitor (-), n=64**	**P-value**
Sex, female	20 (17.7)	7 (14.9)	0.82	1 (5.6)	14 (21.9)	0.17
Age, years	75 (69─80)	76 (69─83)	0.43	70 (68─81)	76 (69─81)	0.49
Pre-stroke mRS score	0 (0─0)	0 (0─1)	0.08	0 (0─1)	0 (0─1)	0.82
Baseline systolic blood pressure, mmHg	162 (140─180)	156 (139─179)	0.57	176 (138─194)	156 (136─180)	0.19
Baseline NIHSS score	14 (10─19)	16 (9─21)	0.80	13 (8─21)	16 (12─23)	0.17
**Medical history**						
Atrial fibrillation	1 (0.9)	4 (8.5)	0.03	1 (5.6)	5 (7.8)	1.00
Hypertension	75 (66.4)	35 (74.5)	0.35	13 (72.2)	36 (56.3)	0.28
Diabetes mellitus	40 (35.4)	14 (29.8)	0.58	4 (22.2)	23 (34.3)	0.40
Dyslipidemia	38 (33.6)	22 (46.8)	0.15	5 (27.8)	19 (29.7)	1.00
Ischemic stroke/TIA prior to index stroke	12 (10.6)	10 (21.3)	0.08	3 (16.7)	10 (15.6)	1.00
Ischemic heart disease	13 (11.5)	4 (8.5)	0.78	2 (11.1)	10 (15.6)	1.00
**Antithrombotic drugs prior to index stroke**						
Single antiplatelet drug	19 (16.8)	11 (23.4)	0.38	3 (16.7)	11 (17.2)	1.00
Dual antiplatelet drugs	4 (3.5)	3 (6.4)	0.42	0 (0.0)	3 (4.7)	1.00
Statin	24 (21.2)	18 (38.3)	0.03	4 (22.2)	17 (26.6)	1.00
**Imaging**						
ASPECTS	8 (6─9)	8 (6─9)	0.82	8 (6─9)	7 (6─9)	0.70
c-ICA occlusion/stenosis			0.47			0.19
c-ICA occlusion	71 (62.8)	33 (70.2)	--	6 (33.3)	33 (51.6)	--
c-ICA stenosis	42 (37.2)	14 (29.8)	--	12 (66.7)	31 (48.4)	--
Degree of stenosis at baseline (NASCET)	100 (95─100)	100 (95─100)	0.50	97.0 (81.4─100.0)	100.0 (80.0─100.0)	0.62
**Distal occluded vessel**			0.91			0.36
Intracranial internal carotid artery	32 (28.3)	13 (27.7)	--	9 (50.0)	18 (28.1)	--

M1 segment of MCA	57 (50.4)	25 (53.2)	--	6 (33.3)	29 (45.3)	--
M2 segment of MCA	24 (21.2)	9 (19.1)	--	3 (16.7)	17 (26.6)	--
**Time delay**						
Time from LKW to hospital arrival, min	131 (69–337)	183 (73–403)	0.58	110 (60–450)	102 (41–220)	0.27
Time from LKW to puncture, min	227 (148–440)	267 (183–504)	0.17	360 (180–882)	166 (125–285)	<0.01
Time from puncture to first mTICI ≥ 2a reperfusion, min	70 (50–110)	83 (48–109)	0.59	55 (33–105)	57 (37–90)	0.99
**Treatment**						
Intravenous thrombolysis	48 (42.5)	13 (27.7)	0.11	4 (22.2)	22 (34.3)	0.40
**Endovascular therapy**						
Stent retriever/combined contact aspiration and stent retriever	5 (4.4)	5 (10.6)	0.16	2 (11.1)	8 (12.5)	1.00
Contact aspiration	6 (5.3)	7 (14.9)	0.06	4 (22.2)	9 (14.1)	0.47
Angioplasty	40 (35.4)	17 (36.2)	1.00	13 (72.2)	42 (65.6)	0.78
Local intraarterial fibrinolysis	1 (0.9)	1 (2.1)	0.50	0 (0.0)	2 (3.1)	1.00
Antegrade thrombectomy	48 (42.5)	16 (34.0)	0.38	8 (44.4)	28 (43.8)	1.00
**Antiplatelet medication in the perioperative period**						
Aspirin	106 (93.8)	14 (29.8)	<0.01	16 (88.9)	9 (14.1)	<0.01
Cilostazol	5 (4.4)	10 (21.3)	<0.01	0 (0.0)	2 (3.1)	1.00
Intravenous ozagrel	3 (2.7)	2 (4.3)	0.63	0 (0.0)	1 (1.6)	1.00

Data are presented as the median (interquartile range) or number (percent).

ASPECTS indicates Alberta Stroke Program Early Computed Tomography Score; c-ICA, cervical internal carotid artery; LKW, last known well; MCA, middle cerebral artery; mRS, modified Rankin Scale; mTICI, modified Thrombolysis In Cerebral Infarction scale; NASCET, North America symptomatic carotid endarterectomy trial; NIHSS, National Institutes of Health Stroke Scale; TIA, transient ischemic attack.

Supplementary Table 4. Outcomes in the patients with and without carotid artery stenting

	CAS (+), n=160				CAS (-), n=82			
	**$P2Y_{12}$ inhibitor (+), n=113**	**$P2Y_{12}$ inhibitor (-), n=47**	**Crude OR (95% CI)**	**Adjusted OR (95% CI)***	**$P2Y_{12}$ inhibitor (+), n=18**	**$P2Y_{12}$ inhibitor (-), n=64**	**Crude OR (95% CI)**	**Adjusted OR (95% CI)***
**Primary outcome**								
Good functional outcome (mRS 0−2 at 90 days)	45 (39.8)	16 (34.0)	1.28 (0.63─2.62)	4.79 (1.19─19.19)	10 (55.6)	20 (31.3)	2.75 (0.94─8.01)	3.56 (0.74─17.13)
**Secondary outcomes**								
Death within 90 days	3 (2.7)	3 (6.4)	0.40 (0.08─2.06)	0.16 (0.01─111.1)	2 (11.1)	8 (12.5)	0.88 (0.17─4.54)	1.13 (0.18─7.18)
mRS score at 90 days	3 (2─4)	3 (2─4)		--	2 (2−4)	4 (2−5)		--
Any hemorrhagic event	27 (23.9)	11 (23.4)	1.03 (0.46─2.29)	0.74 (0.22─2.49)	4 (22.2)	15 (23.4)	0.93 (0.27─3.27)	1.23 (0.32─4.70)
Any ICH	10 (8.8)	8 (17.0)	0.47 (0.17─1.29)	0.56 (0.11─2.87)	2 (11.1)	9 (14.1)	0.76 (0.15─3.90)	0.87 (0.16─4.80)
Symptomatic ICH	5 (4.4)	4 (8.5)	0.50 (0.13─1.94)	0.57 (0.06─5.34)	2 (11.1)	5 (7.8)	1.48 (0.26─8.32)	1.62 (0.26─10.04)

Any ischemic event	10 (8.8)	2 (4.3)	1.29 (0.06–12.46)	1.39 (0.16–11.79)	1 (5.6)	3 (4.7)	1.65 (0.10–21.32)	1.72 (0.15–20.16)
Recurrent ischemic stroke	10 (8.8)	3 (6.4)	1.42 (0.37–5.43)	1.95 (0.23–16.73)	1 (5.6)	6 (9.4)	0.65 (0.12–12.25)	0.65 (0.07–6.22)
Re-occlusion after procedure	4 (3.4)	2 (4.1)	0.83 (0.15–4.67)	1.36 (0.02–112.0)	1 (5.6)	6 (9.4)	0.57 (0.06–5.06)	0.65 (0.07–6.22)
mRS shift (increasing 1 score)	--	--	0.44 (0.18–1.56)	0.56 (0.23–1.33)	8 (38.1)	29 (45.3)	0.66 (0.26–1.68)	0.86 (0.32–2.34)
**Procedural outcomes**								
Final mTICI ≥ 2c reperfusion	109 (96.5)	45 (95.7)	1.21 (0.21–6.84)	0.43 (0.06–33.12)	17 (94.4)	54 (84.4)	3.15 (0.38–26.40)	3.27 (0.36–29.45)
Final mTICI ≥ 2b reperfusion	67 (59.3)	29 (61.7)	0.90 (0.45–1.82)	0.88 (0.29–2.68)	11 (61.1)	29 (45.3)	1.90 (0.65–5.52)	2.26 (0.70–7.27)
Re-occlusion during procedure	11 (9.7)	3 (6.4)	1.58 (0.42–5.95)	0.57 (0.09–3.68)	0 (0.0)	4 (6.3)	--	--

Data are presented as median (interquartile range) or number (percent).

***** Adjusted for sex, age, pre-stroke mRS, baseline NIHSS score, hypertension, diabetes mellitus, dyslipidemia, ASPECTS, intravenous thrombolysis, statin use, and aspirin during the perioperative period

ASPECTS, Alberta Stroke Program Early Computed Tomography Score; CI, confidence interval; ICH, intracranial hemorrhage；mRS, modified Rankin Scale; mTICI, modified Thrombolysis In Cerebral Infarction scale; OR, odds ratio.

Supplementary Table 5. Baseline characteristics between patients with and without $P2Y_{12}$ inhibitor in perioperative aspirin administration

	Aspirin and $P2Y_{12}$ inhibitor (+)*, n=123	Aspirin only or aspirin and APT other than $P2Y_{12}$ inhibitor**, n=23	P-value
Women	19 (15.6)	3 (13.0)	1.00
Age, years	75 (69–80)	76 (67–83)	0.92
Prestroke mRS score	0 (0–0)	0 (0–2)	0.02
Baseline systolic blood pressure, mmHg	163 (142–182)	162 (141–191)	0.71
Baseline NIHSS score	14 (10–20)	18 (8–24)	0.06
**Medical history**			
Atrial fibrillation	2 (1.6)	2 (8.7)	0.12
Hypertension	81 (66.4)	15 (65.2)	1.00
Diabetes mellitus	39 (32.0)	9 (39.1)	0.63
Dyslipidemia	39 (32.0)	6 (26.1)	0.63
Stroke/TIA prior to index stroke	12 (9.8)	4 (17.4)	0.29
Ischemic heart disease	14 (11.5)	4 (17.4)	0.49
**Antithrombotic drugs prior to index stroke**			
Single antiplatelet drug	16 (13.1)	3 (13.0)	1.00
Dual antiplatelet drugs	4 (3.3)	2 (8.7)	0.24
Statin	26 (21.3)	5 (21.7)	1.00
**Imaging**			
ASPECTS	8 (6–9)	7 (6–10)	0.84
c-ICA occlusion/stenosis			1.00
c-ICA occlusion	70 (57.4)	13 (56.5)	--
c-ICA stenosis	52 (42.6)	10 (43.5)	--
Degree of stenosis at baseline (NASCET)	100 (95–100)	100 (95–100)	0.72
**Distal occluded vessel**			0.79

Intracranial internal carotid artery	38 (31.1)	6 (26.1)	--
M1 segment of MCA	60 (49.2)	13 (56.5)	--
M2 segment of MCA	24 (19.7)	4 (17.4)	--
**Time delay**			
Time from LKW to hospital arrival, min	125 (68–358)	247 (77–705)	0.22
Time from LKW to puncture, min	230 (150–445)	280 (177–761)	0.34
Time from puncture to first mTICI ≥ 2a reperfusion, min	69 (48–110)	66 (40–93)	0.29
**Treatment**			
Intravenous thrombolysis	50 (41.0)	5 (21.7)	0.10
**Endovascular therapy**			
Stent retriever/combined contact aspiration and stent retriever	6 (4.9)	2 (8.7)	0.61
Contact aspiration	10 (8.2)	2 (8.7)	1.00
Angioplasty	50 (41.0)	15 (65.2)	0.05
Carotid artery stenting	106 (86.9)	14 (60.9)	<0.01
Local intraarterial fibrinolysis	1 (0.8)	0 (0.0)	1.00
Antegrade thrombectomy	51 (41.8)	9 (39.1)	1.00
**Antiplatelet medication during the perioperative period**			
Cilostazol	4 (3.3)	11 (47.8)	<0.01
Intravenous ozagrel	3 (2.5)	1 (4.3)	0.50

Data are presented as the median (interquartile range) or number (percent).

*Of the 123 patients, all three drugs (aspirin, $P2Y_{12}$ inhibitor, and cilostazol) were administered during surgery in 4 patients (3.3%).

** Of the 23 patients taking aspirin alone or aspirin and an APT other than a $P2Y_{12}$ inhibitor, 12 (52.2%) were taking aspirin alone, and 11 (47.8%) were taking aspirin and cilostazol.

ASPECTS indicates Alberta Stroke Program Early Computed Tomography Score; c-ICA, cervical internal carotid artery; LKW, last known well; MCA, middle cerebral artery; mRS, modified Rankin Scale; mTICI, modified Thrombolysis In Cerebral Infarction scale; NASCET, North America symptomatic carotid endarterectomy trial; NIHSS, National Institutes of Health Stroke Scale; TIA, transient ischemic attack.

Supplementary Table 6. Outcomes in patients with and without a P2Y12 inhibitor who received aspirin in the perioperative period

	Aspirin and $P2Y_{12}$ inhibitor (+)*, n=123	Aspirin only or aspirin and APT other than $P2Y_{12}$ inhibitor**, n=23	Crude OR (95% CI)	Adjusted OR (95% CI)***
**Primary outcome**				
Good functional outcome (mRS 0–2 at 90 days)	50 (41.0)	2 (8.7)	7.29 (1.64–32.50)	6.71 (1.27–35.48)
**Secondary outcomes**				
Death within 90 days	4 (3.3)	2 (8.7)	0.36 (0.06–2.07)	0.89 (0.04–20.00)
mRS score at 90 days	3 (2–4)	4 (3–5)	--	--
Any hemorrhagic event	28 (23.0)	9 (39.1)	0.46 (0.18–1.18)	0.38 (0.13–1.10)
Any ICH	11 (9.0)	6 (26.1)	0.28 (0.09–0.86)	0.23 (0.06–0.91)
Symptomatic ICH	6 (4.9)	3 (13.0)	0.34 (0.08–1.49)	0.31 (0.06–1.68)
Any ischemic event	10 (8.2)	2 (8.7)	0.94 (0.19–4.59)	0.52 (0.08–3.21)
Recurrent ischemic stroke	10 (8.2)	2 (8.7)	0.94 (0.19–4.59)	0.63 (0.11–3.77)
Re-occlusion after procedure	5 (4.1)	1 (4.3)	0.94 (0.10–8.44)	0.88 (0.04–18.73)
mRS shift (increasing 1 score)	--	--	0.29 (0.13–0.65)	0.42 (0.17–0.99)
**Procedural outcomes**				
Final mTICI ≥ 2c reperfusion	118 (96.7)	21 (91.3)	2.81 (0.48–16.33)	2.98 (0.29–30.58)
Final mTICI ≥ 2b reperfusion	71 (58.2)	11 (47.8)	1.52 (0.62–3.71)	1.41 (0.53–3.82)
Re-occlusion during procedure	11 (9.0)	4 (17.4)	0.47 (0.14–1.63)	0.27 (0.06–1.17)

Data are presented as median (interquartile range) or number (percent).

*Of the 123 patients, all three drugs (aspirin, $P2Y_{12}$ inhibitor, and cilostazol) were administered during surgery in 4 patients (3.3%).

** Of the 23 patients taking aspirin alone or aspirin and an APT other than a $P2Y_{12}$ inhibitor, 12 (52.2%) were taking aspirin alone, and 11 (47.8%) were taking aspirin and cilostazol.

*****Adjusted for sex, age, pre-stroke mRS, baseline National Institutes of Health Stroke Scale score, hypertension, diabetes mellitus, dyslipidemia, Alberta Stroke Program Early Computed Tomography Score, intravenous thrombolysis, statin use, and angioplasty and carotid artery stenting

CI: confidence interval; ICH, intracranial hemorrhage; IPTW, inverse probability of treatment weighting; mRS, modified Rankin Scale; mTICI, modified Thrombolysis In Cerebral Infarction scale; OR, odds ratio.

Supplementary Table 7. Patient background by APT in the perioperative period

	Not any APT, n=87	SAPT, n=21	DAPT*, n=130	TAPT**, n=4	P-value***
Sex, female	18 (20.7)	2 (9.5)	21 (16.2)	1 (25.0)	0.53
Age, years	76 (70–82)	70 (66–81)	76.00 (69–81)	74 (69–79)	0.58
Pre-stroke mRS score	0 (0–0)	0 (0–1)	0 (0–0)	0 (0–0)	0.47
Baseline systolic blood pressure, mmHg	155 (135–178)	163 (139–183)	163 (140–182)	180 (160–204)	0.17
Baseline NIHSS score	16 (10–21)	17 (11–20)	14 (10–21)	11 (7–12)	0.34
**Medical history**					
Atrial fibrillation	7 (8.0)	0 (0.0)	4 (3.1)	0 (0.0)	0.31
Hypertension	55 (63.2)	15 (71.4)	86 (66.2)	3 (75.0)	0.91
Diabetes mellitus	28 (32.2)	10 (47.6)	43 (33.1)	0 (0.0)	0.31
Dyslipidemia	34 (39.1)	8 (38.1)	41 (31.5)	1 (25.0)	0.68
Stroke/TIA prior to index stroke	15 (17.2)	7 (33.3)	13 (10.0)	0 (0.0)	0.04
Ischemic heart disease	10 (11.5)	3 (14.3)	16 (12.3)	0 (0.0)	0.95
**Antithrombotic drugs prior to index stroke**					
Single antiplatelet drug	18 (20.7)	9 (42.9)	17 (13.1)	0 (0.0)	0.01
Dual antiplatelet drugs	4 (4.6)	0 (0.0)	6 (4.6)	0 (0.0)	0.91
Statin	29 (33.3)	5 (23.8)	28 (21.5)	1 (25.0)	0.25
**Imaging**					
ASPECTS	7 (6–9)	7 (6–8)	8 (6–9)	9 (7–10)	0.35
c-ICA occlusion/ stenosis					0.84
c-ICA occlusion	52 (59.8)	14 (66.7)	75 (57.7)	2 (50.0)	--
c-ICA stenosis	35 (40.2)	7 (33.3)	55 (42.3)	2 (50.0)	--
Degree of stenosis at baseline (NASCET), % (n=99)	100 (80–100)	100 (99–100)	100 (95–100)	92 (76–100)	0.63

**Distal occluded vessel**					0.82
Intracranial internal carotid artery	25 (28.7)	5 (23.8)	40 (30.8)	2 (50.0)	--
M1 segment of MCA	40 (46.0)	11 (52.4)	64 (49.2)	2 (50.0)	--
M2 segment of MCA	22 (25.3)	5 (23.8)	26 (20.0)	0 (0.0)	--
**Time delay**					
Time from LKW to hospital arrival, min	112 (46–241)	139 (49–544)	140 (69–367)	181 (93–3846)	0.17
Time from LKW to puncture, min	220 (135–337)	242 (155–639)	260 (150–465)	264 (186–4569)	0.17
Time from puncture to first mTICI ≥ 2a reperfusion, min	65 (39–106)	69 (47–93)	68 (43–110)	73 (63–102)	0.91
**Treatment**					
Intravenous thrombolysis	29 (33.3)	6 (28.6)	50 (38.5)	2 (50.0)	0.69
**Endovascular therapy**					
Stent retriever/combined contact aspiration and stent retriever	11 (12.6)	2 (9.5)	6 (4.6)	1 (25.0)	0.06
Contact aspiration	14 (16.1)	2 (9.5)	10 (7.7)	0 (0.0)	0.25
Angioplasty	43 (49.4)	12 (57.1)	55 (42.3)	2 (50.0)	0.50
Carotid artery stenting	32 (36.8)	12 (57.1)	112 (86.2)	4 (100.0)	<0.01
Local intraarterial fibrinolysis	3 (3.4)	0 (0.0)	1 (0.8)	0 (0.0)	0.44
Antegrade thrombectomy	35 (40.2)	10 (47.6)	51 (39.2)	4 (100.0)	0.11

Data are presented as the median (interquartile range) or number (percent).

*DAPT was defined as an APT with any two of aspirin, clopidogrel, or cilostazol.

** TAPT was defined as APTs with any three of aspirin, clopidogrel, cilostazol, or prasugrel.

*** Fisher's exact test

APT, antiplatelet therapy; ASPECTS, Alberta Stroke Program Early Computed Tomography Score; c-ICA, cervical internal carotid artery; DAPT, dual antiplatelet therapy; LKW, last known well; MCA, middle cerebral artery; mRS, modified Rankin Scale; mTICI,

modified Thrombolysis In Cerebral Infarction scale; NASCET, North America symptomatic carotid endarterectomy trial; NIHSS, National Institutes of Health Stroke Scale; SAPT, single antiplatelet therapy; TAPT, triple antiplatelet therapy; TIA, transient ischemic attack.

Supplementary Table 8. Details of additional preoperative and perioperative antithrombotic medications

		Additional SAPT				Additional DAPT			
**Antithrombotic medications prior to index stroke**	**Number**	**ASA**	**CLP**	**CSZ**	**PRAS**	**ASA plus CLP**	**ASA plus CSZ**	**ASA plus PRAS**	**CLP plus CSZ**
**None**	180	10	2	13	4	92	6	4	1
**SAPT**									
**ASA**	26	0	6	2	0	9	1	0	0
**CLP**	11	2	0	0	0	5	0	0	0
**CSZ**	2	0	0	0	0	1	0	0	0
**PRAS**	0	0	0	0	0	0	0	0	0
**Oral anticoagulants**									
**Warfarin**	4	0	0	0	0	1	2	0	0
**Dabigatran**	7	0	0	0	0	0	0	0	0
**Rivaroxaban**	2	0	0	0	0	1	0	0	0
**Apixaban**	0	0	0	0	0	0	0	0	0
**Edoxaban**	1	0	0	0	0	0	0	0	0
**Other anticoagulants**	0	0	0	0	0	0	0	0	0
**DAPT**									
**ASA plus CLP**	8	0	0	0	0	4	1	0	0
**ASA plus CSZ**	1	0	0	0	0	0	1	0	0
**ASA plus prasugrel**	0	0	0	0	0	0	0	0	0
**CLP plus CSZ**	0	0	0	0	0	0	0	0	0
**CLP plus PRAS**	1	0	0	0	0	0	0	0	0
**SAPT plus warfarin**	4	0	0	0	0	1	0	1	0

**Single plus direct oral anticoagulant**	4	0	0	0	0	0	2	0	0

Data are presented as numbers.

APT, antiplatelet therapy; ASA, aspirin; CLP, clopidogrel; CSZ, cilostazol; DAPT, dual antiplatelet therapy; PRAS, prasugrel; SAPT, single antiplatelet therapy; TAPT, triple antiplatelet therapy.

Supplementary Table 9. Details of additional preoperative and perioperative antithrombotic doses

	Additional SAPT									Additional DAPT								
**Antithrombotic drugs prior to index stroke**	**ASA**				**CLP**				**PRAS**	**ASA**				**CLP**				**PRAS**
**Doses**	**100**	**162**	**200**	**300**	**75**	**150**	**225**	**300**	**20**	**100**	**162**	**200**	**300**	**75**	**150**	**225**	**300**	**20**
**None**	2	0	8	0	2	0	0	0	0	29	2	52	19	13	1	7	72	4
**SAPT**																		
**ASA**	0	0	0	0	1	0	0	5	0	4	1	4	1	1	0	3	5	0
**CLP**	0	1	0	1	0	0	0	0	0	2	0	2	1	0	0	2	3	0
**CSZ**	0	0	0	0	0	0	0	0	0	0	0	0	1	0	0	1	0	0
**PRAS**	0	0	0	0	0	0	0	0	0	0	0	0	0	0	0	0	0	0
**Oral anticoagulants**																		
**Warfarin**	0	0	0	0	0	0	0	0	0	1	0	2	0	0	0	0	1	0
**Dabigatran**	0	0	0	0	0	0	0	0	0	0	0	0	0	0	0	0	0	0
**Rivaroxaban**	0	0	0	0	0	0	0	0	0	0	0	0	1	0	0	0	1	0
**Apixaban**	0	0	0	0	0	0	0	0	0	0	0	0	0	0	0	0	0	0
**Edoxaban**	0	0	0	0	0	0	0	0	0	0	0	0	0	0	0	0	0	0
**Other anticoagulants**	0	0	0	0	0	0	0	0	0	0	0	0	0	0	0	0	0	0
**DAPT**																		
**ASA plus CLP**	0	0	0	0	0	0	0	0	0	2	0	1	2	2	0	0	2	0
**ASA plus CSZ**	0	0	0	0	0	0	0	0	0	0	0	1	0	0	0	0	0	0
**Aspirin plus PRAS**	0	0	0	0	0	0	0	0	0	0	0	0	0	0	0	0	0	0
**CLP plus CSZ**	0	0	0	0	0	0	0	0	0	0	0	0	0	0	0	0	0	0
**CLP plus PRAS**	0	0	0	0	0	0	0	0	0	0	0	0	0	0	0	0	0	0
**SAPT plus warfarin**	0	0	0	0	0	0	0	0	1	1	0	2	0	0	0	0	1	0

**SAPT plus direct oral anticoagulant**	0	0	0	0	0	0	0	2	0	0	0	0	1	0	0	0	1	0

Data are presented as numbers.

APT, antiplatelet therapy; ASA, aspirin; CLP, clopidogrel; CSZ, cilostazol; DAPT, dual antiplatelet therapy; PRAS, prasugrel; SAPT, single antiplatelet therapy; TAPT, triple antiplatelet therapy.

2 Supplementary Figures

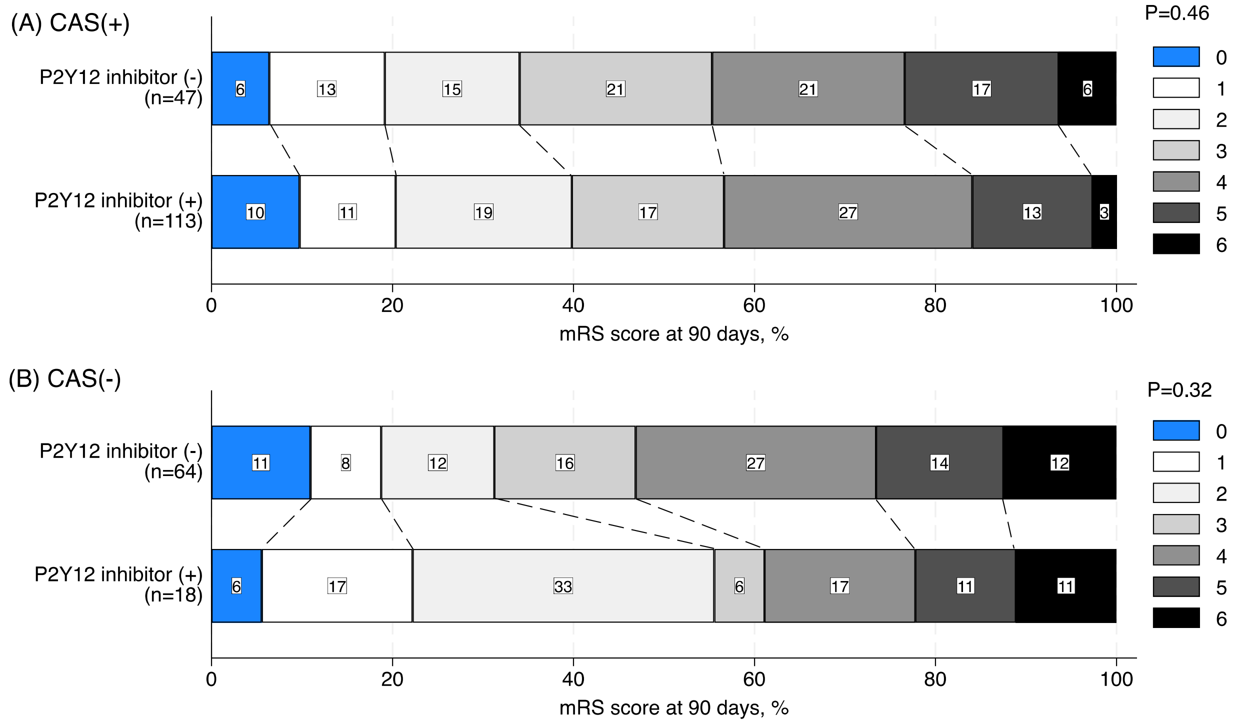

Supplementary Figure 1. Distribution in mRS score at 90 days between patients with and without P2Y12 inhibitors underwent CAS

(**A**) CAS and (**B**) No CAS

CAS, carotid artery stenting; mRS, modified Rankin Scale.

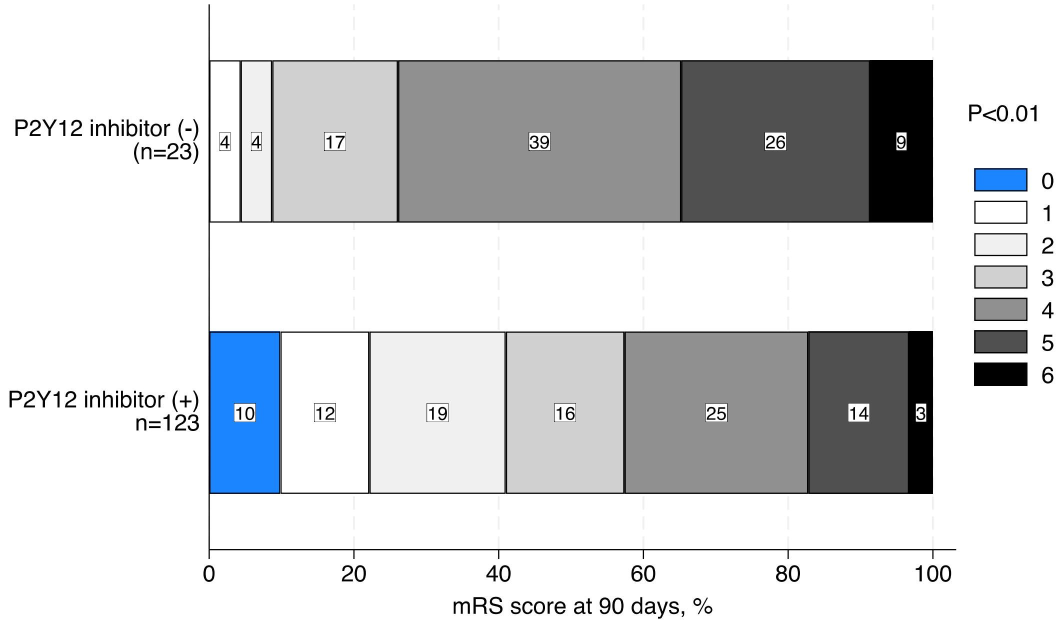

Supplementary eFigure 2. Distribution in mRS score at 90 days in the patients with aspirin in the perioperative period
mRS, modified Rankin Scale.
